# Developmental Validation of the Huaxia Platinum System and application in 3 main ethnic groups of China

**DOI:** 10.1038/srep31075

**Published:** 2016-08-08

**Authors:** Zheng Wang, Di Zhou, Zhenjun Jia, Luyao Li, Wei Wu, Chengtao Li, Yiping Hou

**Affiliations:** 1 Institute of Forensic Medicine, West China School of Preclinical and Forensic Medicine, Sichuan University, Chengdu 610041, China; 2 Shanghai Key Laboratory of Forensic Medicine, Institute of Forensic Science, Ministry of Justice, P.R. China, Shanghai 200063, China; 3 State Key Laboratory of Genetic Engineering, Institute of Genetics, School of Life Sciences, Fudan University, Shanghai 200433, China; 4 Thermo Fisher Scientific Inc., Shanghai 200050, China; 5 Department of Criminal Science and Technology, People’s Public Security University of China, Beijing 100038, China

## Abstract

STRs, scattered throughout the genome with higher mutation rate, are attractive to genetic application like forensic, anthropological and population genetics studies. STR profiling has now been applied in various aspects of human identification in forensic investigations. This work described the developmental validation of a novel and universal assay, the Huaxia Platinum System, which amplifies all markers in the expanded CODIS core loci and the Chinese National Database in one single PCR system. Developmental validation demonstrated that this novel assay is accurate, sensitive, reproducible and robust. No discordant calls were observed between the Huaxia Platinum System and other STR systems. Full genotypes could be achieved even with 250 pg of human DNA. Additionally, 402 unrelated individuals from 3 main ethnic groups of China (Han, Uygur and Tibetan) were genotyped to investigate the effectiveness of this novel assay. The CMP were 2.3094 × 10^−27^, 4.3791 × 10^−28^ and 6.9118 × 10^−27^, respectively, and the CPE were 0.99999999939059, 0.99999999989653 and 0.99999999976386, respectively. Aforementioned results suggested that the Huaxia Platinum System is polymorphic and informative, which provides efficient tool for national DNA database and facilitate international data sharing.

Short tandem repeats (STRs) are DNA regions with a variable number of tandemly repeated short (2–6 bp) sequence motifs^1^. DNA profiling with sets of highly polymorphic autosomal STRs has now been applied in various aspects of human forensics for nearly 30 years^2^,^3^,^4^,^5^. The use of STR profiling to identify perpetrators, massing person, family members and disaster victims has proven to be very successful. In the past few decades, many countries have started to establish forensic DNA databases for the purpose of crime detection^6^,^7^,^8^,^9^. As national DNA databases continue to grow and international cooperation increases, a common set of core loci is required to facilitate data sharing and to minimize adventitious matches.

Initially 13 STR loci were chosen to constitute the Combined DNA Index System (CODIS) by the Federal Bureau of Investigation (FBI) Laboratory, and recently upgraded to 20 loci, i.e. the expanded CODIS core loci^10^,^11^, while a parallel process occurred in European where standardization is based on European Standard Set (ESS)^12^,^13^. All 12 loci in ESS overlap with those in the expanded CODIS core loci (Supplementary Table S1), and several commercial STR kits, such as GlobalFiler PCR Amplification Kit (Thermo Fisher Scientific, MA, USA) and PowerPlex Fusion Systems (Promega, WI, USA), enable robust amplification of these 20 non-redundant loci. To date, a variety of commercial STR kits are chosen in order to cover 20 given STR loci included in the Chinese National Database^14^. However, the harmonization of the expanded CODIS and Chinese National Database has not been achieved yet, 6 non-overlapped loci exist between them (Supplementary Table S1). For STRs to be effective across a wide range of jurisdictions, an all-in-one assay included all non-redundant loci is required.

The Huaxia Platinum System (Thermo Fisher Scientific) was specifically developed to facilitate the processing of reference samples collected for the purpose of database submissions. This system is a 25-locus, six-dye, multiplex that allows co-amplification and fluorescent detection of the 23 loci (CSF1PO, FGA, TH01, TPOX, vWA, D1S1656, D2S1338, D2S441, D3S1358, D5S818, D7S820, D8S1179, D10S1248, D12S391, D13S317, D16S539, D18S51, D19S433, D21S11, D22S1045, D6S1043, Penta D and Penta E) including all the recommended core loci in the expanded CODIS core loci and the Chinese National Database as well as Amelogenin and Y-InDel (rs2032678) for gender determination (More detailed information can be found in Supplementary Table S2). Moreover, it has a new improved PCR buffer formulation and optimized PCR cycling conditions (less than 50 minutes) to enable direct amplification of blood samples. In this study, developmental validation studies of the Huaxia Platinum System were performed following the guidelines of “Validation Guidelines for DNA Analysis Methods (2012)” published by the Scientific Working Group on DNA Analysis Methods (SWGDAM)^15^ and the Chinese National Standard (CNS) “Basic Quality Requirements of Forensic Science Human Fluorescent STR Multiplex PCR Testing Reagent” (GA/T815-2009). Studies of concordance, reproducibility, PCR-based studies, accuracy and precision, stutter analysis, species specificity, sensitivity, mixture, case-type samples testing, stability, and population genetics (3 main ethnic groups of China) were conducted. The validation results demonstrated that the Huaxia Platinum System is robust, specific, sensitive, stable and reliable for human identification applications.

## Results and Discussion

### PCR-based studies

The formulation of the Huaxia Platinum primer set was adjusted to achieve maximal intercolor and intracolor peak height balances for reference samples. The primer set validation experiments showed that full STR profiles were generated at concentrations ranging from −25% (0.75×) to +50% (1.5×) of the optimized formulation; and variations of +25% (1.25×) in the primer set concentration did not impact significantly allele peak height ratio (except for Penta D, peak height ratio = 0.76), intracolor and intralocus peak height balances. However, peak height and balance were changed at 0.75× and 1.5× of the optimized primer set concentration, and allelic drop-out events were observed (D8S1179, D10S1248, Amelogenin and Y-InDel) at −50% (0.5×) primer set concentration (Supplemental Fig. S1). The master mix formulation for the Huaxia Platinum System was optimized to achieve maximal robustness while minimizing both stutter percentages and non-specific amplification. The master mix volume was tested at increments of ±25% and ±50%. The results showed that full profiles were obtained except at 0.5× master mix volume (only CSF1PO, D19S433, Penta E, FGA and D2S1338 generated genotypes). As expected, deviations from the recommended master mix volume gave rise to a drop in average sample peak heights, and intralocus and intercolor peak height balances were affected by 0.75× and 1.5× master mix volume (Supplemental Fig. S2). These results demonstrated that this novel assay could tolerate moderate deviations to the recommended concentration of primer set and master mix.

The Huaxia Platinum System was designed to enable high-throughput processing for databasing laboratories by the minimization of PCR time. With 27 cycles of PCR amplification, the total PCR time was approximately 42 minutes. In the developmental validation study, several thermal cycling parameters were evaluated, including cycle number, denaturing temperature, annealing temperature, extension temperature and final extension time. Generally, the optimal cycle number should generate sufficient peak heights while minimizing the occurrence of off-scale allele peaks and allelic drop-out events. It is critical for each laboratory to determine the optimal cycle number in order to achieve maximal first-pass success and minimize reruns^16^. The cycle number study described in this study included amplifications ranging from 25 to 28 cycles. Full STR profiles were obtained at all cycle numbers tested, and each increase in cycle number led to a corresponding increase in average peak height. Sample peak height, intracolor peak height balances were optimal at the standard 27 cycles, and at 28 cycles several off-scale homozygote allele peaks were observed (Supplemental Fig. S3).

Three alternative denaturation temperatures, 92 °C, 93 °C and 95 °C, were tested against the standard 94 °C. Although minimal effects were observed on overall sample peak height (92 °C), full STR profiles could be obtained at all denaturation temperatures tested (Supplemental Fig. S4). Thermal cyclers could drift over time if not maintained properly and might not always be accurately calibrated, thus annealing temperature studies are important to determine the tolerance levels of an STR multiplex system with slight temperature variation. Annealing temperature studies were conducted by running the amplification protocol with the annealing temperature either one degree above and two degrees below the optimal temperature. No artifact peaks or allelic drop-out events were observed with annealing temperatures between 57 °C and 60 °C. Optimal sample peak heights and intracolor peak height balances were observed at the recommended 59 °C. Increasing the annealing temperature to 60 °C resulted in the suppression of overall sample peak height (Supplemental Fig. S5). The optimal extension temperature maintains the optimal balance between specific amplification of DNA and reproducible intracolor peak height balance. Except for average sample peak heights decreased at 66 °C, full STR profiles were obtained at all extension temperatures tested (63 °C–66 °C) and changes in extension temperature did not show any significant effect on intralocus and intracolor peak height balances (Supplemental Fig. S6). These results demonstrated that the quality and robustness of the Huaxia Platinum System over a number of temperature variables.

Final extension at certain time is significant to ensure complete terminal nucleotide addition. The Huaxia Platinum System yielded incomplete terminal nucleotide addition with less than 5 minutes of final extension at the TPOX, D5S818, vWA, D13S317, and D6S1043 (Supplemental Fig. S7). A final extension greater than 5 min was not detrimental to the assay but provided no benefits. Overall, changes in final extension time did not show any significant effect on sample peak height, intralocus and intracolor peak height balances.

### Concordance, case-type samples and reproducibility

**Figure 1:**
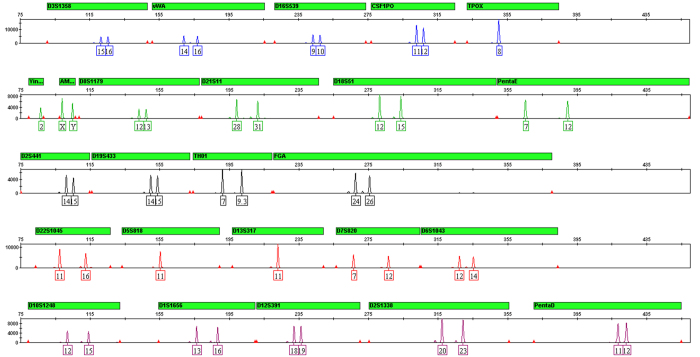
Electropherogram of Control DNA 007 (1 ng) amplified by Huaxia Platinum System. Control DNA 007 was amplified following the recommended protocol (27 cycles). Amplified product was separated on an Applied Biosystems 3500 Genetic Analyzer. Panel labeled “Yin…” is Y-InDel. Panel labeled “AM…” is Amelogenin.

Concordance among commercial STR systems is essential to maintain the relevance and accuracy of data generated with the Huaxia Platinum System. Control DNA 007 (Thermo Fisher Scientific) and 202 Han samples were chosen for concordance study. Combined STR loci in GlobalFiler PCR Amplification Kit and GoldenEye 20A kit (Peoplespot, Beijing, China) could obtain 23 autosomal loci in Huaxia Platinum System. All genotyping was performed with GeneMapper1 ID-X v1.4 software (Thermo Fisher Scientific) using bins, panels and allelic ladders manufacturer provided. The results showed that the STR genotypes generated by Huaxia Platinum System were in 100% concordance with those by combined two kits. The representative electropherogram of Control DNA 007 is shown in Fig. 1.

The ability to obtain reliable results from typically encountered case-type samples and reproducibility between laboratories could indicate whether this novel assay would be widely applied to the field of forensic science. Control DNA 007 and the prepared 30 case-type samples were tested in two different accredited laboratories. All samples gave complete and concordant genotypes with direct (blood samples) or normal amplification in two laboratories, indicating that the Huaxia Platinum System has good reproducibility between laboratories and is suitable for typically encountered biological materials in forensic laboratories. The electropherogram of one human bone sample DNA (1.0 ng) which was amplified with this novel assay is shown in Supplemental Fig. S8.

### Accuracy, precision, and stutter studies

Determining sizing precision and accuracy includes evaluation of measurement error and assessing performance for reliable and accurate genotyping^16^. 202 Han samples were used to measure the deviation of each sample allele size from the corresponding allelic ladder allele size. All sample alleles tested were within ±0.50 bp (mostly within ±0.30 bp) of the corresponding allele in the allelic ladder. The results indicated that this novel assay could reliably detect the genotypes and could accurately determine the microvariant.

Allelic ladder comprise the most prevalent alleles at the 23 STRs, Amelogenin and Y-InDel in main populations of China, optimized by the manufacturer. In brief, PCR products of different genotypes at each STR marker were cloned in plasmid, and the successful clones of each allele were diluted, mixed and balanced to produce a single allelic ladder for each STR. Then, allelic ladders of different STR were mixed and balanced in appropriate portions. The representative electropherogram of allelic ladder is shown in Supplemental Fig. S9. Allelic ladder sizing precision was calculated from multiple injections (16 times) of allelic ladder. The standard deviation (SD) of the mean of allele size measurements was less than 0.15 bp (Supplemental Fig. S10). The result demonstrated that this novel assay could reliably and accurately determine the genotypes.

Stutter products are natural result of strand slippage during PCR amplification^17^. A minor peak with one repeat unit in length smaller (minus stutter) or larger (plus stutter) than the associated allele is commonly observed (n ± 5 for pentanucleotide STR marker, n ± 4 for tetranucleotide STR marker, n ± 3 for trinucleotide STR marker). Percent stutter was calculated based on 202 Han samples, and the stutter average and SD are shown in Table 1. All loci showed a trend of increasing stutter percentages with increasing allele size, which was consistent with prior observations^16^,^18^. Notably, stutter percentages of pentanucleotide STR markers in this novel assay were slightly higher than previous reports^14^,^19^, reminding the manufacturer of further optimization. The stutter filter was determined using the mean stutter percentages plus three SD values, which could be used as a guideline for whether a peak was a stutter peak or minor component concealed in a mixture.

### Sensitivity and stability studies

**Figure 2:**
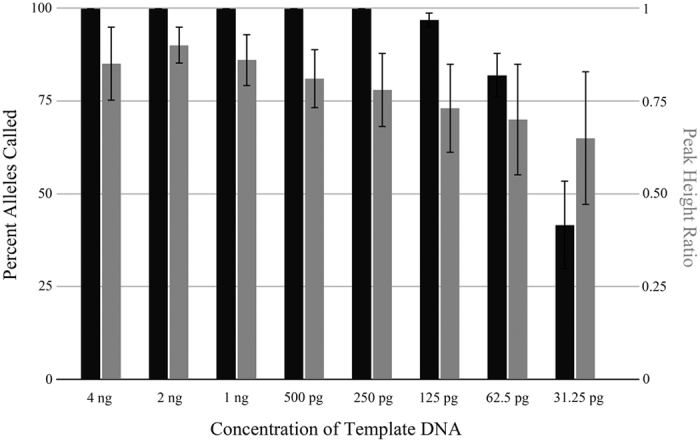
Sensitivity test of template DNA ranging from 4 ng to 31.25 pg. The percentage of alleles called (black) and peak height ratio (gray) are shown. Error bars represent the standard deviation between all replicates.

Sensitivity testing was helpful for determining the lower and upper limits of the STR system with peak heights above the analysis threshold on a Genetic Analyzer using the injection conditions specified for the cycling conditions used. In this study, serial dilutions of the Control DNA 007 were analyzed on an Applied Biosystems 3500 Genetic Analyzer (Thermo Fisher Scientific). For allele designation and peak height ratio determination a 175 RFU threshold was used. Results showed that the optimal mass of template DNA for Huaxia Platinum System ranged from 2 ng to 250 pg. When the template DNA increased to 4 ng, full genotypes of STR loci were obtained with the average peak height of 19550 RFUs and appearance of bleed-through at homozygous loci (some peak heights above 30000 RFUs), which were likely to lead to confusing results. Conversely, when reduced to 125 pg, the average percentage profile was 96.8% with some peak heights below 175 RFUs or dropout. To continue decreasing the mass of DNA template, Fig. 2 indicates that the average percentage profiles decreased sharply and peak height imbalance increased. At 62.5 pg and 31.25 pg 81.9% and 41.6% of alleles were respectively called, which were not suitable for genotyping.

The ability of Huaxia Platinum System to obtain results from DNA recovered from two PCR inhibitors was evaluated in this stability testing. The results showed that this assay could tolerate hematin and humic acid in certain ranges. Typically, the large amplicons were the first to drop out. Supplemental Fig. S11 illustrates that average percentage of loci detected was decreased while the concentrations of PCR inhibitor increased. Full profiles were obtained with hematin concentration <400 μM and humic acid <60 ng/μL.

Forensic samples might be exposed to several unfavorable environmental factors. Artificial degraded DNA samples by digesting at different time point were tested to determine the amplification efficiency of degraded samples. Consistent with prior observations^20^, the reduction of peak heights and allele numbers began with larger amplicons. For Control DNA 007 that had been incubated for 1 min, complete profiles were obtained with the reduction of average peak heights for all loci compared to the positive control (1 ng of undigested Control DNA 007). For 2 min incubation, one allele (allele 12 at Penta D) dropout was observed in two profiles, and the other profile was complete. For 4 min incubation, genotypes with large amplicons could not be obtained (Supplemental Fig. S12).

### Mixture studies

**Figure 3:**
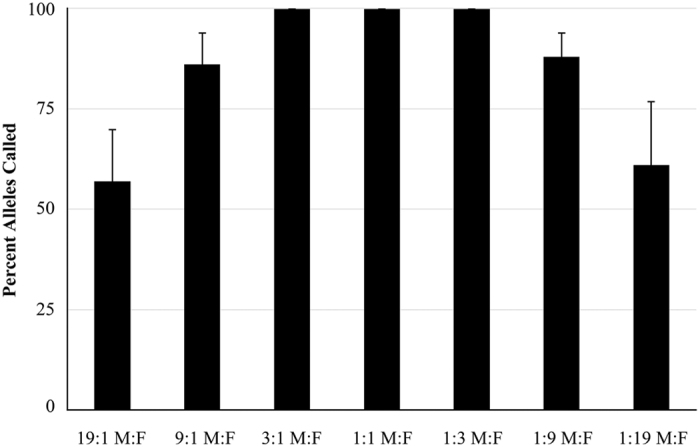
Mixture analysis. A two-source DNA mixture was created at various ratios (1 ng total DNA) and amplified with Huaxia Platinum System using the recommend protocol. The percentage of the minor alleles detected at a given mixture ratio is shown. Error bars represent the standard deviation between all replicates.

Evidence samples that contain biological materials originating from more than one individual are commonly encountered in forensic casework. Mixture studies can assist to mixture interpretation, including the number of contributors, the minor and major contributor profiles, and contributor proportions or ratios. The genotypes of the Control DNA 007 (male, M) and 9947A (female, F) are shown in Supplementary Table S3. A summary of all data generated can be seen in Fig. 3. As the value of mixture ratios increased, there was a decrease in the percentage of minor component that could be identified. The minor component of 3:1 and 1:3 mixture ratios were able to type robustly (100% calling rate). At 9:1 and 1:9 ratios, minor component yielded partial profiles (86% and 88% calling rate) because they were below the detection threshold (175 RFUs). However at 19:1 and 1:19 ratios, 57% and 61% alleles were respectively called since the minor component (50 pg) was too low to detect.

### Species specificity

DNA samples in forensic laboratories may contain trace or even excess non-human DNA; thus, various types of non-human DNA were tested to evaluate any PCR products of this novel assay. Despite being run at an amount much higher (10 ng) than what is recommended for extracted DNA, none of the microbes tested yielded any peaks above the 175 relative fluorescence units (RFUs) threshold. For vertebrate DNA sources, 8 of 17 samples were amplified to varying extends, producing fragments in or between calling region. The findings for DNA sources that yielded a peak above the 175 RFU threshold are summarized in Table 2. All non-specific amplification peaks are shown in Supplemental Fig. S13. At most, mouse DNA showed reproducible peaks above 175 RFU at three loci: D2S441, D19S433 and TH01. Nevertheless, these non-specific amplification peaks are distinguishable from human profiles by their low peak height, number of off-ladder peaks and the irregular and imbalanced nature of amplification. Therefore, this novel system has good species specificity and is suitable for human identity testing.

### Genetic parameters of the Huaxia Platinum System

As the world’s most populous country, China has some distinct ethnic groups which vary in culture and social customs. A cost-efficient assay should show highly informative among populations. This study is focused on 3 main ethnic groups, including Han and 2 main minority ethnic groups (Uygur and Tibetan). The Han population, as the biggest ethnic group, constitutes about 92% of the total population in China. The 2 minority ethnic groups are mainly located in Northwest of China, and both of them are regarded as typical examples of Chinese ethnic minorities. A total of 402 bloodstains from above 3 ethnic groups were surveyed.

Forensic parameters including observed heterozygosity (H_o_), expected heterozygosity (H_e_), polymorphism information content (PIC), power of discrimination (PD), power of exclusion (PE) and typical paternity index (TPI) for each locus in Han, Uygur and Tibetan population are shown in Supplementary Table S4–6, respectively. No significant deviations from Hardy-Weinberg equilibrium were observed for any of 23 autosomal loci or in any of the ethnic groups after Bonferroni correction (p > 0.002174), and no significant deviations from linkage disequilibrium between pairwise STR loci after Bonferroni correction (p > 0.0001976) in 3 ethnic groups (Supplementary Table S7–9).

In the Han population, high value of H_e_ (>0.7), PD (>0.9) and PIC (>0.7) was observed at each STR locus except for CSF1PO, D3S1358, TH01, TPOX and D10S1248. Compared to Penta E locus showing the most variation with 22 alleles observed, TPOX showed the lowest amount of variation with 5 alleles observed. The three most PD loci were Penta E, D2S1338 and D6S1043, and the combined match probability (CMP) value was 2.3094 × 10^−27^. The highest and lowest PE loci were D6S1043 (0.8177) and TH01 (0.2957), respectively, and the combined power of exclusion (CPE) value was 0.99999999939059. Similar high CMP (4.3791 × 10^−28^ and 6.9118 × 10^−27^) and CPE (0.99999999989653 and 0.99999999976386) were observed in Uygur and Tibetan, respectively.

Allelic frequencies of the 23 loci among 3 ethnic groups are listed in Supplementary Table S10. The exact test of population differentiation was performed between Han and 2 minority ethnic groups. As shown in Supplementary Table S11, unlike the X-STRs^21^ and Y-STRs^22^,^23^, no significant difference was observed between Han and the other 2 groups at all 23 autosomal STR loci after Bonferroni correction (p > 0.001087). Aforementioned results suggested that the Huaxia Platinum System is polymorphic, informative and universal, and suitable for forensic individual identification, paternity or kinship investigations and DNA databasing.

## Conclusions

In this paper, the developmental validation of a novel STR system, the Huaxia Platinum System, and the application information in 3 main ethnic groups of China are reported. 23 autosomal STRs including all the recommended core loci in the expanded CODIS core loci and the Chinese National Database as well as Amelogenin and Y-InDel (rs2032678) were co-amplified in a single PCR reaction system. The studies of developmental validation demonstrated that the panel is an accurate, sensitive, robust and universal tool. The statistical parameters of forensic importance for this novel system in Han, Uygur and Tibetan populations showed high polymorphism and universality for human identification purposes. Also, the Huaxia Platinum System provides the capability for national and international databases sharing of DNA profiles.

## Methods

### Ethics Statement

Human blood samples were collected upon approval of the Ethics Committee at the Institute of Forensic Medicine, Sichuan University. Informed consent was obtained from each participant. All the methods were carried out in accordance with the approved guidelines of Institute of Forensic Medicine, Sichuan University. This study was approved by the Ethics Committee of Sichuan University.

### Sample preparation

402 peripheral blood samples were collected from 202 unrelated Han Chinese (101 females and 101 males) recruited from Sichuan Province, 100 unrelated Uygur Chinese (50 females and 50 males) recruited from Xinjiang Uygur Autonomous Region, and 100 unrelated Tibetan Chinese (54 females and 46 males) recruited from Tibet Autonomous Region. Human genomic DNA was extracted using QIAamp DNA Blood Mini Kit (Qiagen, Hilden, Germany) according to the manufacturer’s instructions. The quantity of the DNA template was determined using Quantifiler Human DNA Quantification Kit on the 7500 Real-time PCR System (Thermo Fisher Scientific).

A set of 30 case-type samples, including 5 blood stains, 5 muscle samples, 5 buccal swabs, 5 hair rooted samples, 4 semen stains, 3 cigarette butts and 3 bone samples, was collected and prepared by Department of Criminal Science and Technology, People’s Public Security University of China. Bloodstains were prepared for direct amplification by spotting 75 μL of whole blood onto the center of the sampling area on Whatman FTA cards (GE Healthcare Life Sciences, NJ, USA; 3 samples) and sterile filter papers (Sangon Biotech, Shanghai, China; 2 samples). Human genomic DNA of other 25 case-type samples was extracted using PrepFiler Express BTA Forensic DNA Extraction Kit on AutoMate Express Forensic DNA Extraction System (Thermo Fisher Scientific), and quantified using Quantifiler Human DNA Quantification Kit on 7500 Real-time PCR System.

Purified DNA of species samples, including common animal species (cat, dog, chicken, pig, mouse, rat, rabbit, goose, horse, goat, donkey, duck, sheep, pigeon, deer, woodchuck and turtle) and microorganisms (Escherichia coli, Pseudomonas aeruginosa, Saccharomyces cerevisiae and Streptococcus salivarius), was used to assess species specificity of this novel assay.

Typical control DNA of 007 and 9947A human cell line samples (Thermo Fisher Scientific) were used as positive samples for PCR and electrophoresis. Control DNA 007 was also applied to concordance, reproducibility, PCR-based studies, sensitivity, stability and mixture study.

### Pre-PCR sample preparation

Prior to PCR amplification, blood on plain paper (untreated paper substrates, sterile filter paper in this study) must be lysed efficiently. 1.2 mm discs punched from these paper substrates were treated with 5 μL of Prep-N-Go lysis buffer (Thermo Fisher Scientific) for cell lysis. For chemically treated paper substrates (FTA cards in this study) that contain compounds that can lyse cells upon contact, one−1.2 mm disk punched from substrates can be amplified directly without additional sample preparation.

### PCR amplification and genotyping

PCR amplification was performed with 27 PCR cycles according to the manufacturer’s protocol on a ProFlex PCR System (Thermo Fisher Scientific). The PCR system was a 25 μL reaction volume containing 10 μL of master mix, 10 μL of primer set and 1 ng of template DNA or Prep-N-Go buffer (for blood on filter paper) or Low TE Buffer (for blood on FTA cards). The standard thermal cycling conditions consisted of an initial step at 95 °C for 1 min; followed by 27 cycles of 94 °C for 3 s, 59 °C for 16 s, and 65 °C for 29 s; and a final extension at 60 °C for 5 min.

Amplification products were separated and detected on the Applied Biosystems 3500 Genetic Analyzers using POP-4 polymer and 36 cm capillary array. One microlitre of PCR amplified sample or allelic ladder was added to a mixture containing 9.6 μL of deionized Hi-Di formamide and 0.4 of μL GeneScan 600 LIZ Size Standard v2.0 (Thermo Fisher Scientific). The mixture was injected at 1.2 kV for 16 s and electrophoresed at 13 kV for 1550 s with a run temperature at 60 °C. Initial fragment sizing and allele calling were performed using GeneMapper ID-X v.1.4 software with the peak amplitude threshold set at 175 RFUs for all colors.

### Primer set and master mix concentration

The Huaxia Platinum System was designed to enable fast and robust amplification from single source samples, which has a new improved PCR formulation and optimized PCR cycling conditions. To evaluate the performance of primer set and to assess the reliability and robustness of the master mix formulation, 1 ng of Control DNA 007 was amplified in triplicate at the standard primer mix (or master mix) concentration and at increments of ± 25% and ± 50% volume added into the PCR reaction.

### NThermal cycling parameters

The thermal cycling parameters were evaluated to establish the optimal performance window of amplification for the Huaxia Platinum System. Control DNA 007 was tested in triplicate at each varied thermal cycling parameter: 1. Cycle number: 25, 26, 27 (standard) and 28 cycles; 2. Denaturing temperature: 92 °C, 93 °C, 94 °C (standard) and 95 °C; 3. Annealing temperature: 57 °C, 58 °C, 59 °C (standard) and 60 °C; 4. Extension temperature: 63 °C, 64 °C, 65 °C (standard) and 66 °C; 5. Final extension time: 1, 2, 5 (standard) and 8 min.

### Concordance, case-type samples and reproducibility

A concordance study of the Huaxia Platinum System is essential to evaluate the accuracy of allelic designations. Control DNA 007 and DNA extracted from 202 Han samples were typed with GlobalFiler PCR Amplification Kit and the GoldenEye 20A kit for concordance testing. Combined STR loci in these two kits can obtain 23 autosomal loci in Huaxia Platinum System. PCR amplification was conducted according to the respective protocol on a ProFlex PCR System, and genotyping was performed with GeneMapper ID-X v.1.4 software using corresponding allelic ladders, panels and bins.

A set of 30 case-type samples was tested in two accredited laboratories (Forensic Genetics Laboratory, Sichuan University; Forensic Genetics Laboratory, People’s Public Security University of China) to evaluate the ability to obtain reliable genotypes from those typically encountered forensic case-type samples and to detect the accuracy and reproducibility.

### Accuracy, precision, and stutter studies

Sizing accuracy is defined as the deviation in size of each sample allele from the corresponding allelic ladder allele^16^. 202 Han samples were amplified using the standard PCR condition at 27 cycles to measure sizing accuracy. Allelic ladder sizing precision was assessed by calculating the standard deviation in the size values obtained from multiple repeated injections (n = 16) on an Applied Biosystems 3500 Genetic Analyzer.

Stutter products are amplicons that are typically one repeat smaller or larger than the true allele and arise during PCR because of strand slippage. The proportion of the stutter product (minus and plus stutter) relative to the main allele was measured by dividing the height of the stutter peak by the height of the true allele peak. The stutter analysis of this novel assay was performed according to the results obtained from the genotyping of 202 Han samples. For stutter calculation, saturating allele peaks were removed, and the minimum threshold for the stutter peak height was set at 20 RFUs.

### Sensitivity and Stability studies

To demonstrate the sensitivity of the Huaxia Platinum System, a series of dilutions of Control DNA 007 (4 ng, 2 ng, 1 ng, 500 pg, 250 pg, 125 pg, 62.5 pg and 31.25 pg) was tested in triplicate. All samples were amplified in a total reaction volume of 25 μL.

Stability studies were conducted to determine the ability of the Huaxia Platinum System for obtaining results from compromised samples and digested DNA. One nanogram of Control DNA 007 containing different concentrations of two kinds of common forensic inhibitors: humic acid (Sigma–Aldrich Corporation, MO, USA) and hematin (Sigma–Aldrich Corporation) were prepared. The concentrations evaluated here were 20 ng/μL, 40 ng/μL, 60 ng/μL, 80 ng/μL, 100 ng/μL, 150 ng/μL of humic acid and 100 μM, 200 μM, 400 μM, 600 μM, 800 μM of hematin. Artificial degraded DNA by incubating Control DNA 007 with DNase I for different time point was also tested in this study. Artificial degraded DNA was prepared by incubating 5 ng Control DNA 007 in a 10 μL reaction with 1 μL corresponding 10 × DNase I buffer and 2U DNase I (Takara, Dalian, China) at 37 °C for 1, 2 and 4 min. These treated samples were amplified with the Huaxia Platinum System in triplicate at 27 PCR cycles.

### NMixture study

Forensic sample analysis usually involves mixture materials that contain the DNA from more than one individual. Mixture study was performed with mixed DNA samples with known ratios of DNA. Mixture samples were prepared using two Control DNA (Control DNA 007: 9947 A DNA) with a total DNA input of 1.0 ng and the following ratios were tested: 19:1, 9:1, 3:1, 1:1, 1:3, 1:9 and 1:19. The genotypes of the Control DNA 007 and 9947A were shown in Supplementary Table S3. Two accredited laboratories (Forensic Genetics Laboratory, Sichuan University; Forensic Genetics Laboratory, People’s Public Security University of China) evaluated the ability to resolve minor contributor alleles with the Huaxia Platinum System.

### Species specificity

The species specificity of the Huaxia Platinum System was evaluated by a series of tests on non-human DNA to see if other biological samples mixed with the human DNA would affect final amplification result. DNA samples (10 ng) from 17 common animal species (cat, dog, chicken, pig, mouse, rat, rabbit, goose, horse, goat, donkey, duck, sheep, pigeon, deer, woodchuck and turtle), and common microorganisms (Escherichia coli, Pseudomonas aeruginosa, Saccharomyces cerevisiae and Streptococcus salivarius) were run in PCR amplification reactions with the Huaxia Platinum System in triplicate at 27 PCR cycles.

### Population studies

In order to evaluate the forensic efficiency of this novel assay for application in 3 main ethnic groups of China, genotype data of 402 unrelated individuals including 202 Han, 100 Uygur and 100 Tibetan were analyzed.The observed heterozygosity (H_o_) and expected heterozygosity (H_e_) were estimated using Arlequin v3.5^24^. The exact test of Hardy-Weinberg equilibrium (HWE) and linkage disequilibrium (LD) were also performed using Arlequin v3.5. Forensic parameters were determined by calculating polymorphism information content (PIC), power of discrimination (PD), power of exclusion (PE) and typical paternity index (TPI) using PowerStats V12 spreadsheet (Promega)^25^.

### Quality control

Control DNA 007 and ddH_2_O were used as positive and negative controls respectively for each batch of genotyping. The main experiments were conducted at the Forensic Genetics Laboratory of Institute of Forensic Medicine, Sichuan University, which is an accredited laboratory (ISO 17025), in accordance with quality control measures. We strictly followed the recommendations of Chinese National Standards and Scientific Working Group on DNA Analysis Methods (SWGDAM)^15^.

## Additional Information

**How to cite this article**: Wang, Z. *et al*. Developmental Validation of the Huaxia Platinum System and application in 3 main ethnic groups of China. *Sci. Rep.* **6**, 31075; doi: 10.1038/srep31075 (2016).

## Supplementary Material

Supplementary Information

## Figures and Tables

**Table 1:** Stutter analysis of the Huaxia Platinum System.

Locus	Count	Mean (%)	Standard deviation	Mean ± 3SD
FGA	137	8.12	1.09	11.38
TH01	85	5.69	1.57	10.41
vWA	92	4.25	2.36	11.33
D1S1656	73	3.82	1.77	9.13
D2S441	68	2.27	1.28	6.12
D3S1358	106	3.07	1.84	8.60
D8S1179	145	3.81	2.39	10.99
D10S1248	81	3.04	2.43	10.33
D12S391	109	5.93	2.61	13.76
D18S51	127	6.24	1.66	11.23
D21S11	115	3.77	2.17	10.27
D22S1045	108	2.91	1.45	7.26
CSF1PO	86	4.83	1.96	10.72
TPOX	121	4.35	1.71	9.49
D2S1338	155	8.18	1.15	11.62
D5S818	98	5.30	1.64	10.22
D7S820	133	6.32	2.07	12.52
D13S317	89	6.19	1.85	11.75
D16S539	107	5.74	2.95	14.58
D19S433	93	5.27	2.88	13.92
D6S1043	148	7.91	3.27	17.71
Penta D	165	6.04	3.18	15.57
Penta E	179	6.91	2.33	13.90

**Table 2:** Summary of animal DNA reactivity with the Huaxia Platinum System.

DNA source	Artifact size(s)	Allele call
Dog	~180 bp (FAM), ~348 bp (VIC)	17 in vWA, OMR (Outside Marker Range)
Chicken	~328 bp (TAZ)	12 in D6S1043
Pig	~227 bp (FAM)	OL (Off-Ladder) in D16S539
Goose	~334 bp (FAM)	OL in D2S1338
Goat	~285 bp (VIC), ~436 bp (VIC)	29 in D2S11, OL in Penta E
Sheep	~109 bp (TAZ), ~112 bp (TAZ), ~310 bp (TAZ)	15 and 16 in D22S1045, OL in D6S1043
Mouse	~101 bp (NED), ~146 bp (NED), ~178 bp (NED)	14 in D2S411. 13 in D19s433, OL in TH01
Rat	~125 bp (FAM), ~299 bp (FAM), ~307 bp (FAM)	16 in D3S1358, 10 and 12 in CSF1PO
