# Supplementary material for: Developmental Validation of the Huaxia Platinum System and application in 3 main ethnic groups of China
Source: Sci Rep. 2016 Aug 8;6:31075. doi: 10.1038/srep31075 (PMC4976323; doi:10.1038/srep31075)
Supplement: Supplementary Information [file srep31075-s1.pdf]

Supplementary Figures S1-S13 and Supplementary Tables S1-S11

Developmental Validation of the Huaxia Platinum System and application in 3 main ethnic groups of China

Zheng Wang[1,2,3], Di Zhou[4], Zhenjun Jia[5], Luyao Li[1], Wei Wu[4], Chengtao Li[2], Yiping Hou[1*]

[1] Institute of Forensic Medicine, West China School of Preclinical and Forensic Medicine, Sichuan University, Chengdu 610041, China

[2] Shanghai Key Laboratory of Forensic Medicine, Institute of Forensic Science, Ministry of Justice, P.R. China, Shanghai 200063, China

[3] State Key Laboratory of Genetic Engineering, Institute of Genetics, School of Life Sciences, Fudan University, Shanghai 200433, China

[4] Thermo Fisher Scientific Inc., Shanghai 200050, China

[5] Department of Criminal Science and Technology, People's Public Security University of China, Beijing 100038, China

[*] Corresponding author: Yiping Hou

Institute of Forensic Medicine, West China School of Preclinical and Forensic Medicine, Sichuan University, Chengdu 610041, China

E-mail: forensic@scu.edu.cn; Phone: +86-28-85501550; Fax: +86-28-85501549.

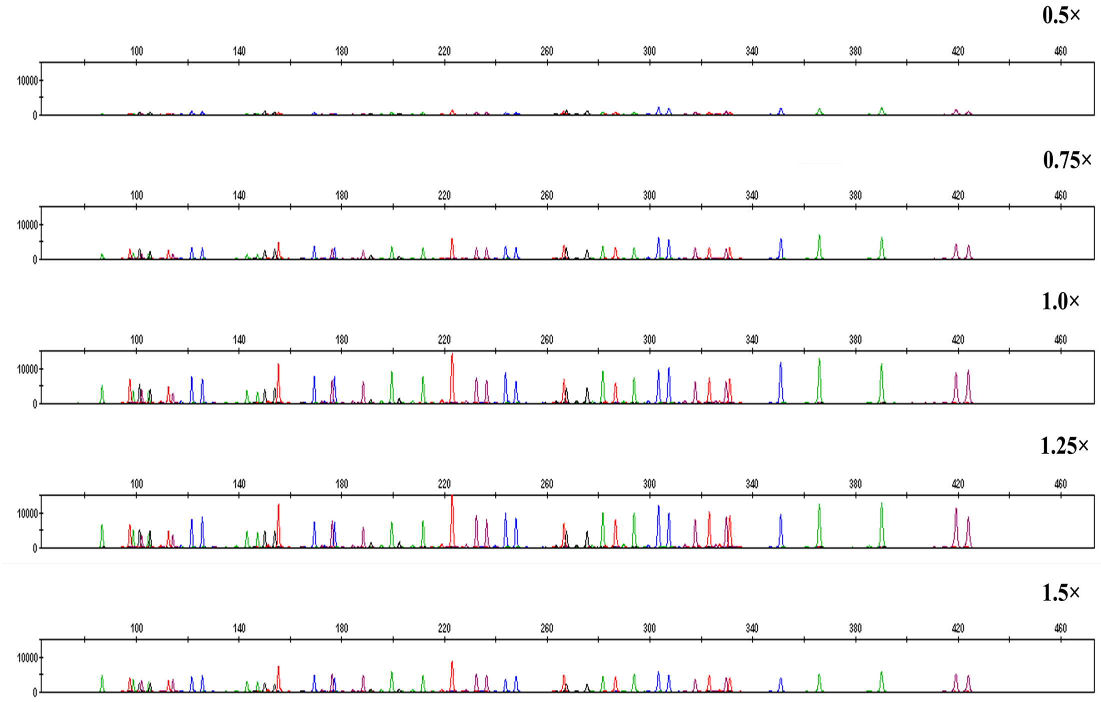

Supplementary Fig. S1. Effect of Huaxia Platinum primer concentration on human DNA amplification. Five concentrations were examined: 0.5×, 0.75×, 1.0×, 1.25× and 1.5× (top to bottom, respectively).

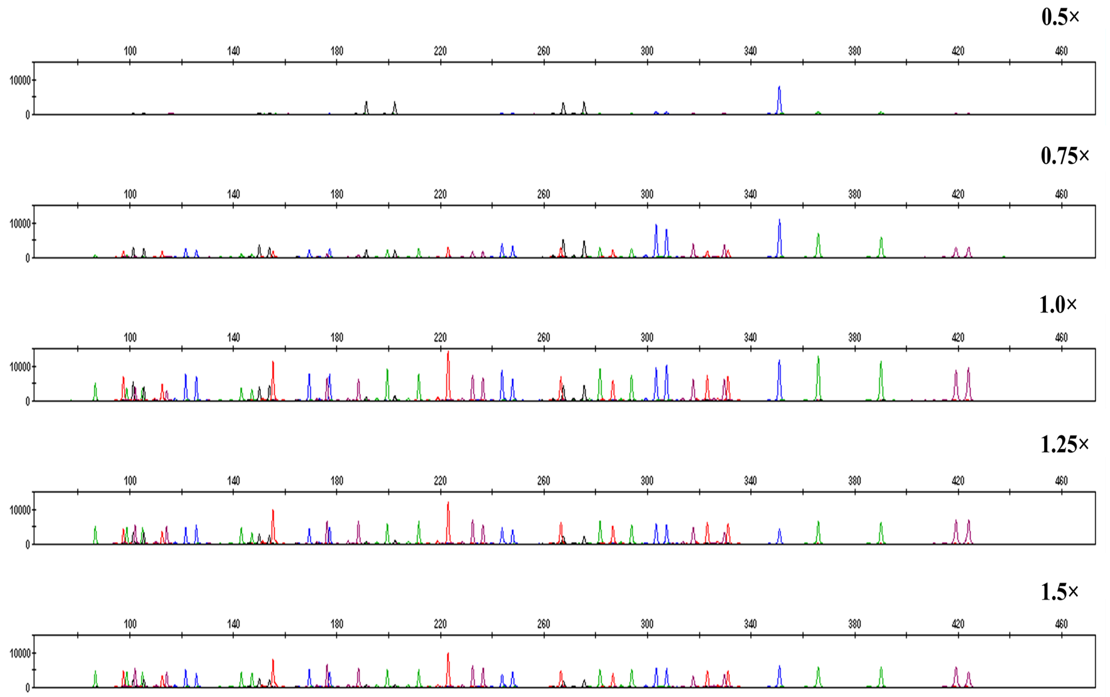

Supplementary Fig. S2. Effect of Huaxia Platinum master mix concentration on human DNA amplification. Five concentrations were examined: 0.5×, 0.75×, 1.0×, 1.25× and 1.5× (top to bottom, respectively).

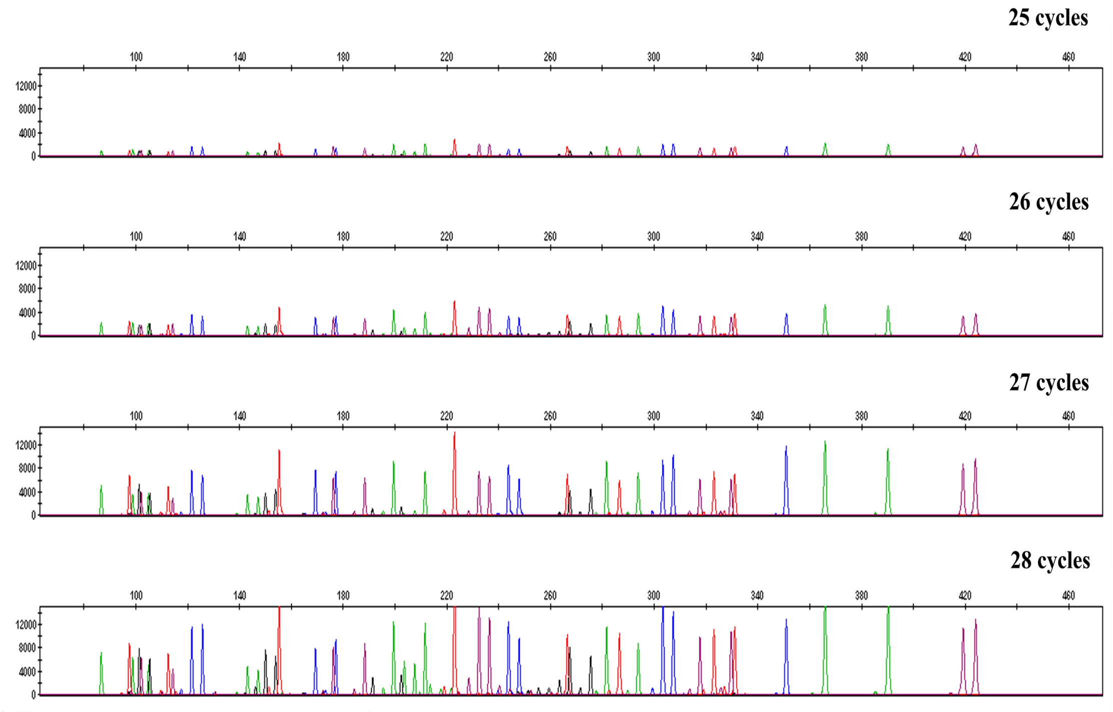

Supplementary Fig. S3. Cycle number analysis of the Huaxia Platinum System on human DNA amplification. Four cycle numbers were examined: 25, 26, 27 and 28 cycles (top to bottom, respectively).

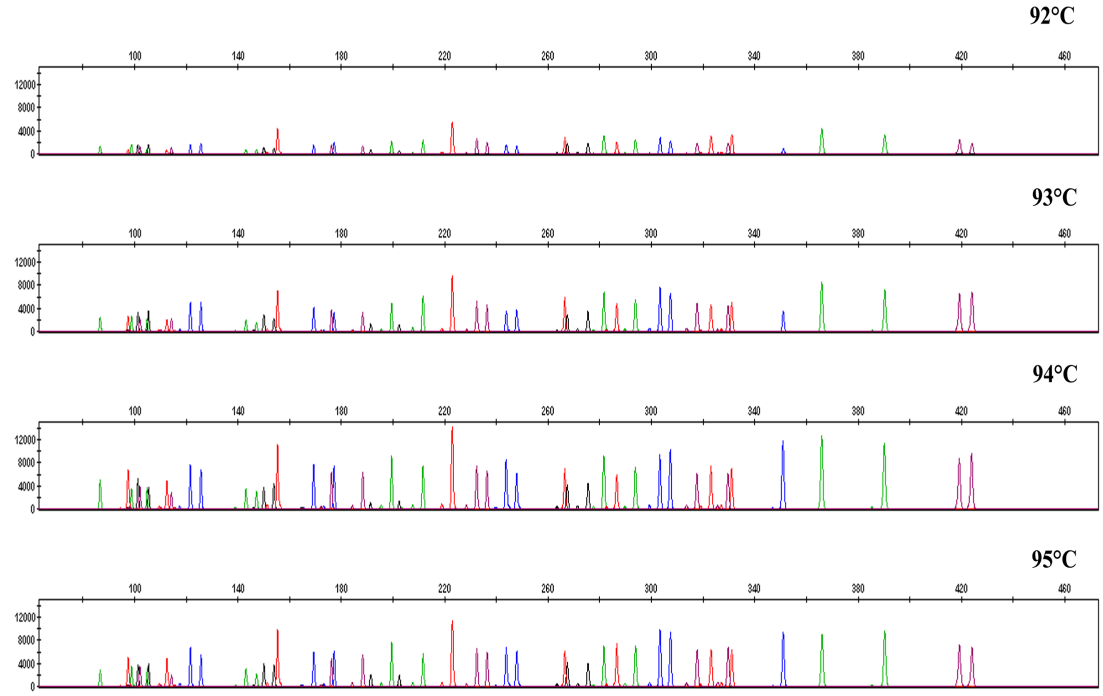

Supplementary Fig. S4. Effect of different denaturation temperatures on human DNA amplification. Four temperatures were examined: 92 °C, 93 °C, 94 °C and 95 °C (top to bottom, respectively).

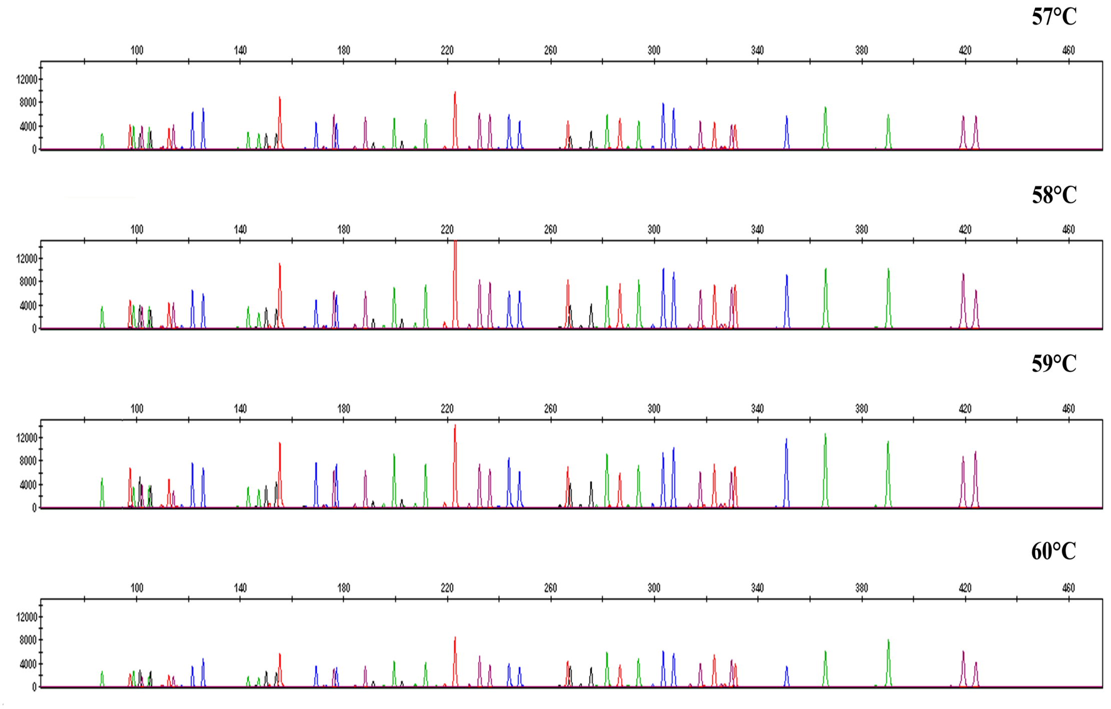

Supplementary Fig. S5. Effect of different annealing temperatures on human DNA amplification. Four temperatures were examined: 57 °C, 58 °C, 59 °C and 60 °C (top to bottom, respectively).

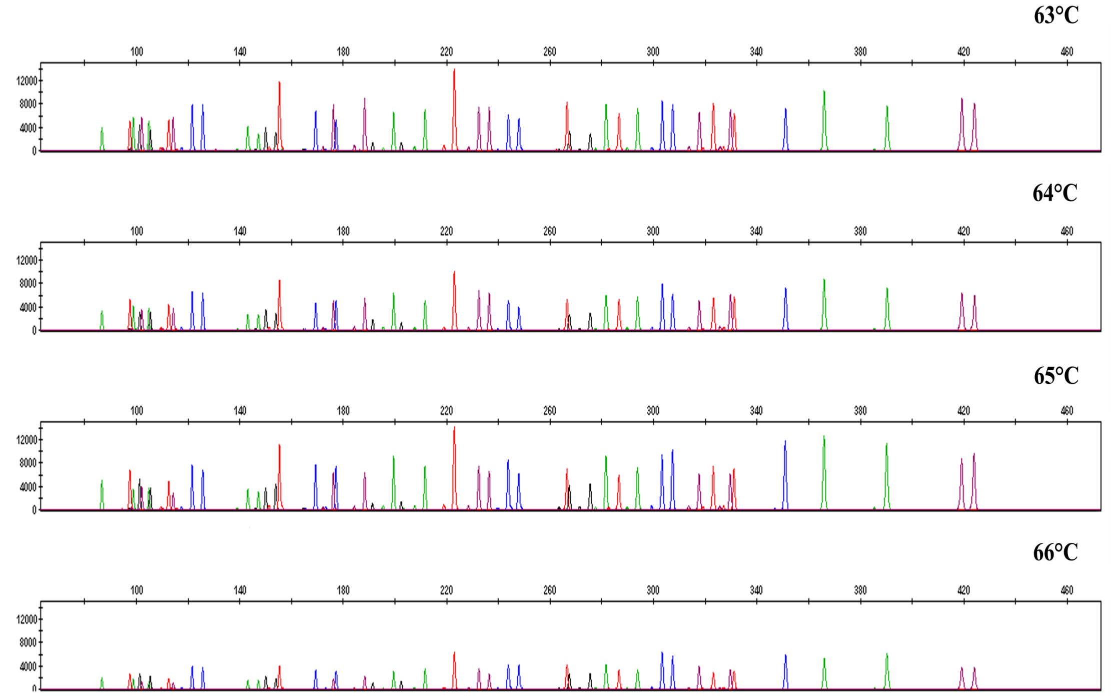

Supplementary Fig. S6. Effect of different extension temperatures on human DNA amplification. Four temperatures were examined: 63 °C, 64 °C, 65 °C and 66 °C (top to bottom, respectively).

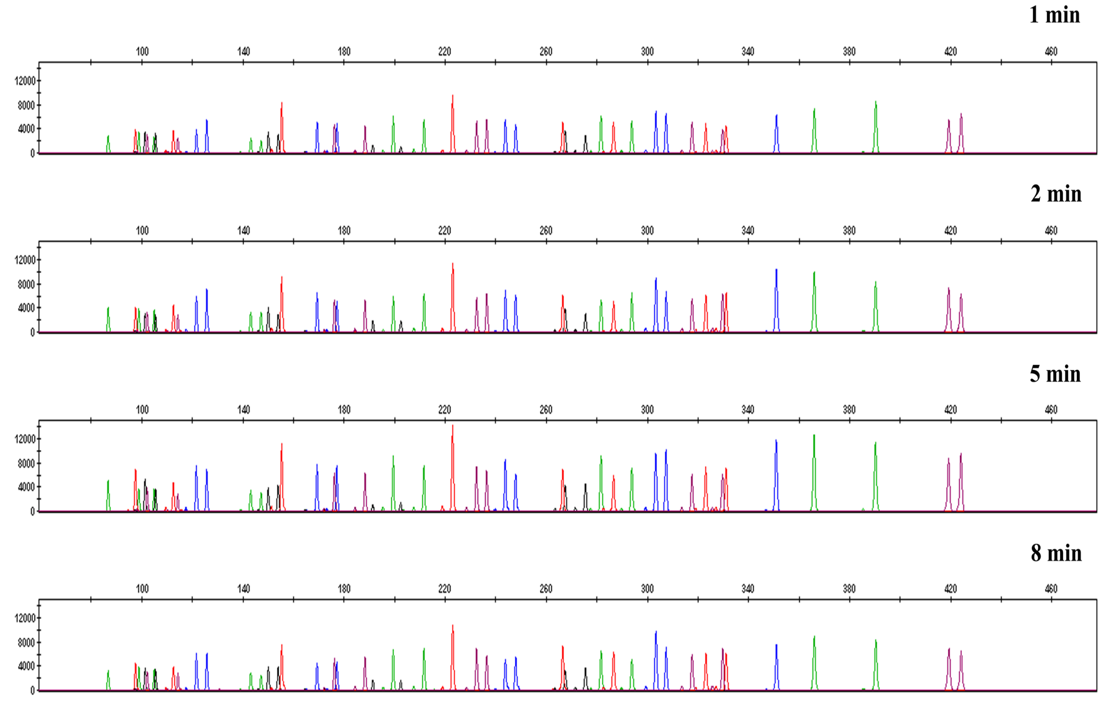

Supplementary Fig. S7. Effect of different final extension times on human DNA amplification. Four final extension times were examined: 1 min, 2 min, 5 min and 8 min (top to bottom, respectively).

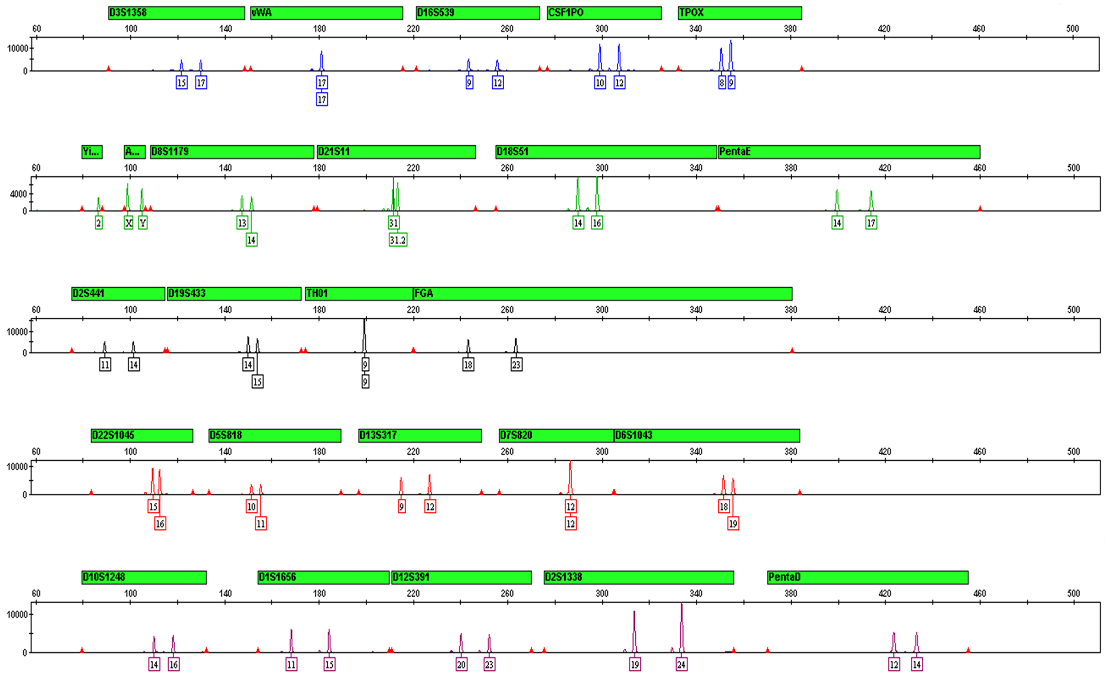

Supplementary Fig. S8. Electropherogram of one human bone sample (approximately 1 yeaar) DNA amplified by the Huaxia Platinum System.

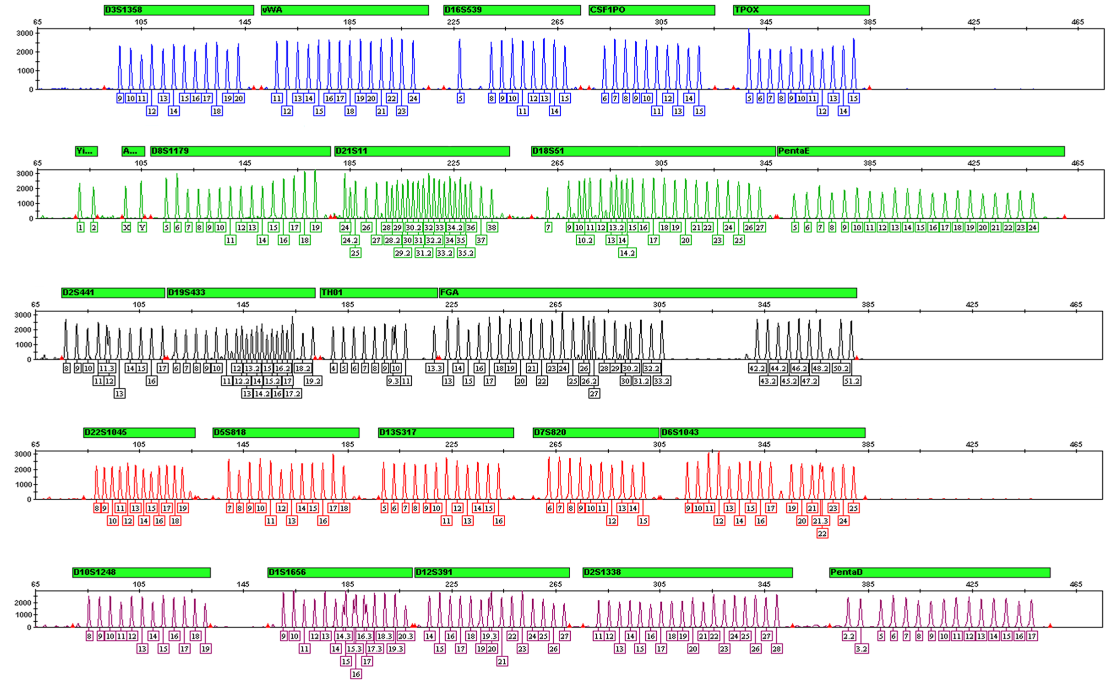

Supplementary Fig. S9. Electropherogram of the allelic ladder designed for the Huaxia Platinum System.

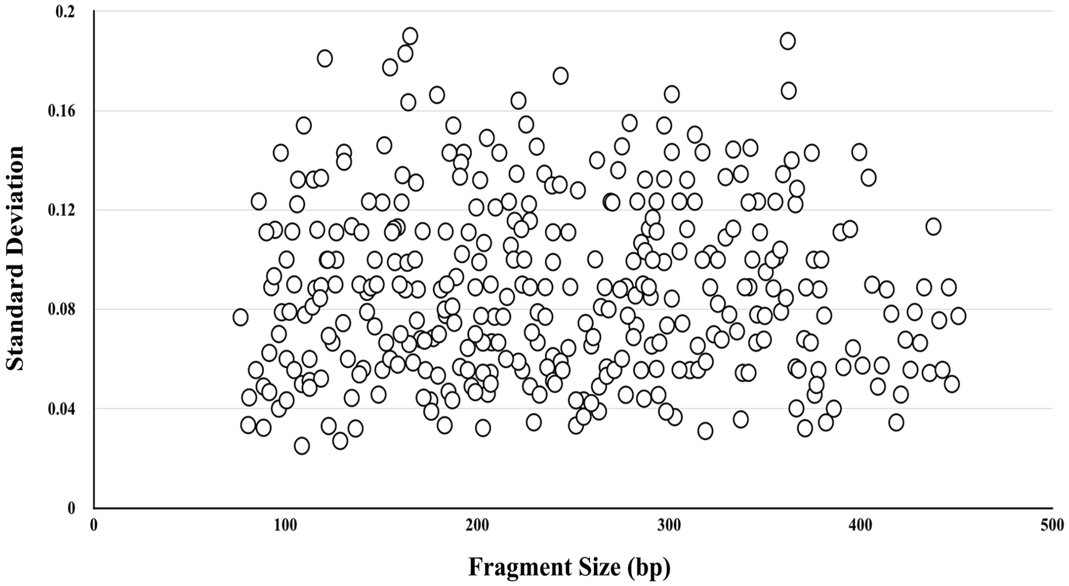

Supplementary Fig. S10. Sizing variation of allelic ladders on 3500 Genetic Analyzer (n = 16). The X-axis represents the size length of the allelic ladders, while the Y-axis stands for the variation of the standard deviation corresponding to each allelic ladder.

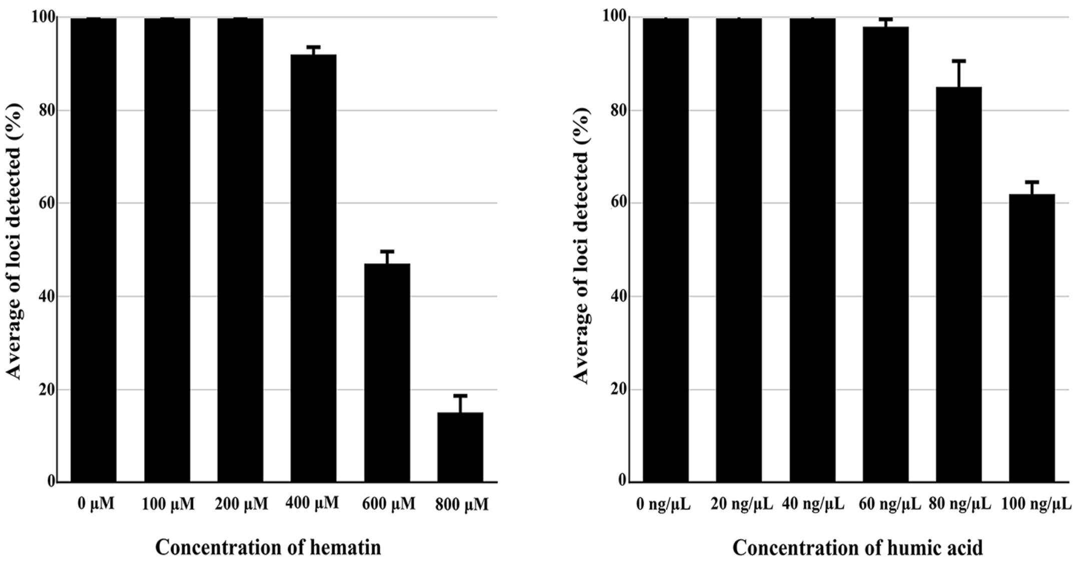

Supplementary Fig. S11. Stability testing of two PCR inhibitors. Average of loci detected by amplifying 1 ng of control DNA 007 with the Huaxia Platinum System in the presence of increasing amounts of PCR inhibitors.

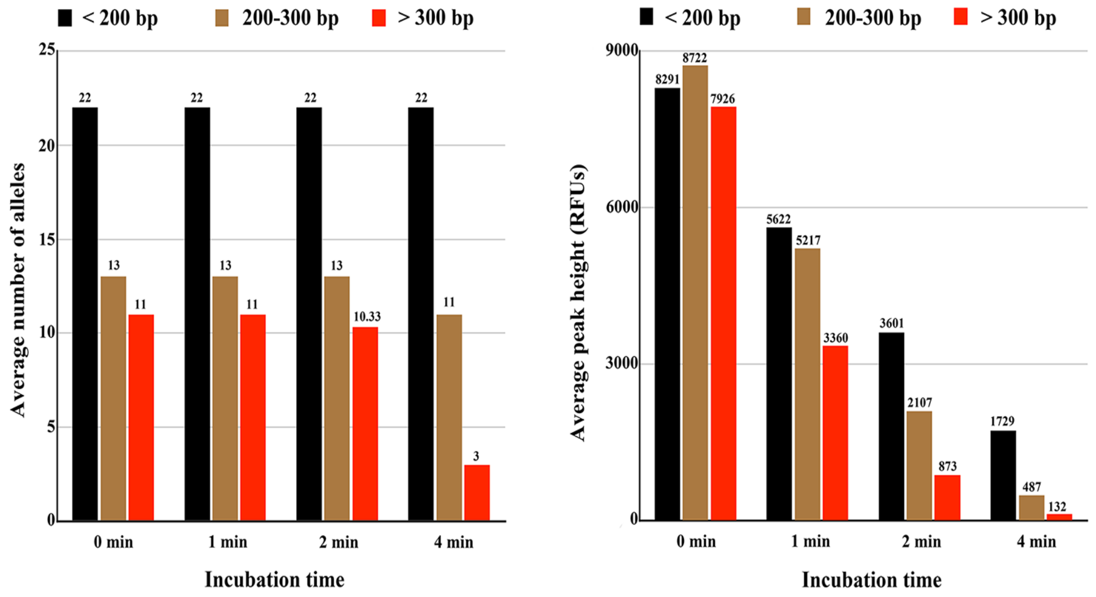

Supplementary Fig. S12. The detection information (the average number of alleles and the average peak height) of artificial degraded DNA by digesting at different time points.

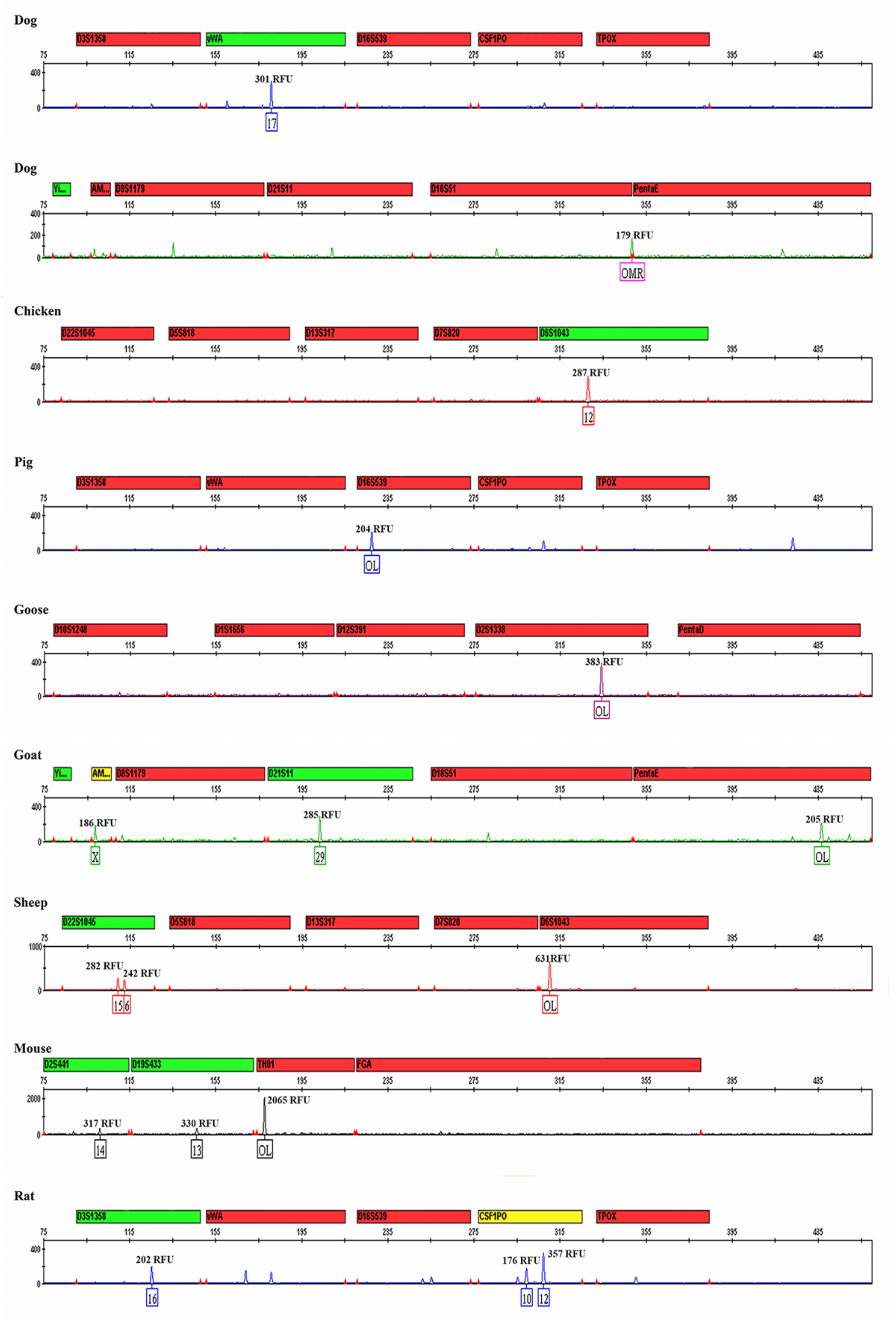

Supplementary Fig. S13. A representative electropherogram for each DNA source which yielded a peak above the 175 RFU threshold.

Supplementary Table S1. Recommended core loci of three Database and the loci in the Huaxia Platinum System.

Locus	Expanded CODIS core loci	ESS	Chinese National Database	Huaxia Platinum System
FGA	✓	✓	✓	✓
TH01	✓	✓	✓	✓
vWA	✓	✓	✓	✓
D1S1656	✓	✓	✓	✓
D2S441	✓	✓		✓
D3S1358	✓	✓	✓	✓
D8S1179	✓	✓	✓	✓
D10S1248	✓	✓		✓
D12S391	✓	✓	✓	✓
D18S51	✓	✓	✓	✓
D21S11	✓	✓	✓	✓
D22S1045	✓	✓		✓
CSF1PO	✓		✓	✓
TPOX	✓		✓	✓
D2S1338	✓		✓	✓
D5S818	✓		✓	✓
D7S820	✓		✓	✓
D13S317	✓		✓	✓
D16S539	✓		✓	✓
D19S433	✓		✓	✓
D6S1043			✓	✓
Penta D			✓	✓
Penta E			✓	✓

Supplementary Table S2. General information of the Huaxia Platinum System.

Locus	Chromosomal Location	Alleles included in Allelic Ladder	Dye label	Control DNA 007 Genotype
D3S1358	3p21.31	9, 10, 11, 12, 13, 14, 15, 16, 17, 18, 19, 20	6-FAM™	I5, 16
vWA	12p13.31	11, 12, 13, 14, 15, 16, 17, 18, 19, 20, 21, 22, 23, 24	6-FAM™	14, 16
D16S539	16q24.1	5, 8, 9, 10, 11, 12, 13, 14, 15	6-FAM™	9, 10
CSF1PO	5q33.1	6, 7, 8, 9, 10, 11, 12, 13, 14, 15	6-FAM™	11, 12
TPOX	2p25.3	5, 6, 7, 8, 9, 10, 11, 12, 13, 14, 15	6-FAM™	8, 8
Y indel	Yq11.221	1, 2	VIC™	2
Amelogenin	X: p22.1-22.3<br>Y: p11.2	X, Y	VIC™	X, Y
D8S1179	8q24.13	5, 6, 7, 8, 9, 10, 11, 12, 13, 14, 15, 16, 17, 18, 19	VIC™	I2, 13
D21S11	21q21.1	24, 24.2, 25, 26, 27, 28, 28.2, 29, 29.2, 30, 30.2, 31, 31.2, 32, 32.2, 33, 33.2, 34, 34.2, 35, 35.2, 36, 37, 38	VIC™	28, 31
D18S51	18q21.33	7, 9, 10, 10.2, 11, 12, 13, 13.2, 14, 14.2, 15, 16, 17, 18, 19, 20, 21, 22, 23, 24, 25, 26, 27	VIC™	12, 15
Penta E	15q26.2	5, 6, 7, 8, 9, 10, 11, 12, 13, 14, 15, 16, 17, 18, 19, 20, 21, 22, 23, 24	VIC™	7, 12
D2S441	2p14	8, 9, 10, 11, 11.3, 12, 13, 14, 15, 16, 17	NED™	14, 15
D19S433	19q12	6, 7, 8, 9, 10, 11, 12, 12.2, 13, 13.2, 14, 14.2, 15, 15.2, 16, 16.2, 17, 17.2, 18.2, 19.2	NED™	14, 15
TH01	11p15.5	4, 5, 6, 7, 8, 9, 9.3, 10, 11, 13.3	NED™	7, 9.3
FGA	4q28	13, 14, 15, 16, 17, 18, 19, 20, 21, 22, 23, 24, 25, 26, 26.2, 27, 28, 29, 30, 30.2, 31.2, 32.2, 33.2, 42.2, 43.2, 44.2, 45.2, 46.2, 47.2, 48.2, 50.2, 51.2	NED™	24, 26
D22S1045	22q12.3	8, 9, 10, 11, 12, 13, 14, 15, 16, 17, 18, 19	TAZ™	11, 16
D5S818	5q23.2	7, 8, 9, 10, 11, 12, 13, 14, 15, 16, 17, 18	TAZ™	11, 11
D13S317	13q31.1	5, 6, 7, 8, 9, 10, 11, 12, 13, 14, 15, 16	TAZ™	11, 11
D7S820	7q21.11	6, 7, 8, 9, 10, 11, 12, 13, 14, 15	TAZ™	7, 12
D6S1043	6q15	9, 10, 11, 12, 13, 14, 15, 16, 17, 19, 20, 21, 21.3, 22, 23, 24, 25	TAZ™	12, 14
D10S1248	10q26.3	8, 9, 10, 11, 12, 13, 14, 15, 16, 17, 18, 19	SID™	12, 15
D1S1656	1q42	9, 10, 11, 12, 13, 14, 14.3, 15, 15.3, 16, 16.3, 17, 17.3, 18.3, 19.3, 20.3	SID™	13, 16
D12S391	12p13.2	14, 15, 16, 17, 18, 19, 19.3, 20, 21, 22, 23, 24, 25, 26, 27	SID™	18, 19
D2S1338	2q35	11, 12, 13, 14, 15, 16, 17, 18, 19, 20, 21, 22, 23, 24, 25, 26, 27, 28	SID™	20, 23
Penta D	21q22.3	2.2, 3.2, 5, 6, 7, 8, 9, 10, 11, 12, 13, 14, 15, 16, 17	SID™	11, 12

Supplementary Table S3. The genotypes of Control DNA 007 and 9947A were used in mixture studies.

Locus	Control DNA 007	9947A
D3S1358	15/16	14/15
vWA	14/16	17/18
D16S539	9/10	11/12
CSF1PO	11/12	10/12
TPOX	8/8	8/8
Y indel	2	/
Amelogenin	X/Y	X/X
D8S1179	12/13	13/13
D21S11	28/31	30/30
D18S51	12/15	15/19
Penta E	7/12	12/13
D2S441	14/15	10/14
D19S433	14/15	14/15
TH01	7/9.3	8/9.3
FGA	24/26	23/24
D22S1045	11/16	11/14
D5S818	11/11	11/11
D13S317	11/11	11/11
D7S820	7/12	10/11
D6S1043	12/14	12/18
D10S1248	12/15	13/15
D1S1656	13/16	18.3/18.3
D12S391	18/19	18/20
D2S1338	20/23	19/23
Penta D	11/12	12/12

Supplementary Table S4. Forensic parameters for 23 STRs in HAN population (N=202).

Locus	H_o	H_e	p-value	PIC	PD	PE	TPI
D3S1358	0.7376	0.7250	0.2887	0.6748	0.8658	0.4888	1.9057
vWA	0.7574	0.7965	0.7731	0.7626	0.9285	0.5226	2.0612
D16S539	0.7574	0.7803	0.1937	0.7448	0.9165	0.5226	2.0612
CSF1PO	0.6584	0.7367	0.2041	0.6892	0.8870	0.3739	1.4583
TPOX	0.6683	0.6244	0.8524	0.5656	0.7970	0.3810	1.5075
D8S1179	0.8218	0.8458	0.2710	0.8244	0.9543	0.6401	2.8056
D21S11	0.7821	0.8089	0.0205	0.7821	0.9313	0.5664	2.2955
D18S51	0.8366	0.8439	0.9603	0.8235	0.9538	0.6687	3.0606
Penta E	0.9001	0.9173	0.3907	0.9085	0.9826	0.8076	5.3158
D2S441	0.8168	0.7905	0.8741	0.7572	0.9216	0.6306	2.7297
D19S433	0.7970	0.8202	0.8981	0.7954	0.9459	0.5935	2.4634
TH01	0.6040	0.6584	0.4393	0.6112	0.8405	0.2957	1.2625
FGA	0.8465	0.8558	0.7582	0.8379	0.9625	0.6880	3.2581
D22S1045	0.7822	0.7584	0.1711	0.7149	0.8922	0.5664	2.2955
D5S818	0.7673	0.7794	0.3041	0.7440	0.9164	0.5399	2.1489
D13S317	0.7574	0.8064	0.7168	0.7766	0.9353	0.5236	2.0612
D7S820	0.8267	0.7744	0.7177	0.7399	0.9081	0.6496	2.8857
D6S1043	0.9109	0.8707	0.6184	0.8547	0.9627	0.8177	5.6111
D10S1248	0.7574	0.7299	0.1450	0.6872	0.8771	0.5226	2.0612
D1S1656	0.7970	0.7941	0.8265	0.7683	0.9334	0.5935	2.4634
D12S391	0.8416	0.8367	0.2294	0.8146	0.9475	0.6880	3.2581
D2S1338	0.8762	0.8750	0.0407	0.8597	0.9635	0.7472	4.0400
Penta D	0.7772	0.8088	0.7022	0.7824	0.9374	0.5575	2.2444

Supplementary Table S5. Forensic parameters for 23 STRs in Uygur population (N=100).

Locus	H_o	H_e	p-value	PIC	PD	PE	TPI
D3S1358	0.7600	0.7786	0.3291	0.7391	0.9116	0.5270	2.0833
vWA	0.8600	0.8209	0.1023	0.7912	0.9282	0.7147	3.5714
D16S539	0.8500	0.8092	0.9206	0.7771	0.9288	0.6949	3.3333
CSF1PO	0.7500	0.7234	0.9572	0.6704	0.8648	0.5098	2.0000
TPOX	0.6500	0.6671	0.4609	0.6129	0.8370	0.3552	1.4286
D8S1179	0.8700	0.8347	0.7753	0.8089	0.9394	0.7346	3.8462
D21S11	0.8600	0.8417	0.6240	0.8194	0.9464	0.6949	3.3333
D18S51	0.9000	0.8728	0.1356	0.8543	0.9574	0.7954	5.0000
Penta E	0.9000	0.9299	0.0597	0.9202	0.9794	0.7954	5.0000
D2S441	0.7600	0.7779	0.3114	0.7436	0.9154	0.5270	2.0833
D19S433	0.7200	0.8305	0.1952	0.8063	0.9466	0.4599	1.7857
TH01	0.8100	0.7802	0.9665	0.7409	0.9094	0.6177	2.6316
FGA	0.7600	0.8439	0.1643	0.8197	0.9496	0.5270	2.0833
D22S1045	0.8000	0.7526	0.6752	0.7056	0.8808	0.5990	2.5000
D5S818	0.7300	0.7631	0.3375	0.7214	0.9006	0.4762	1.8519
D13S317	0.8200	0.7995	0.3519	0.7665	0.9216	0.6367	2.7778
D7S820	0.7700	0.80840	0.7873	0.7757	0.9324	0.5446	2.1739
D6S1043	0.8700	0.8639	0.6555	0.8445	0.9604	0.7346	3.8462
D10S1248	0.8100	0.7712	0.7864	0.7297	0.9010	0.6177	2.6316
D1S1656	0.8200	0.8526	0.5272	0.8329	0.9554	0.6367	2.7778
D12S391	0.8500	0.8722	0.6899	0.8547	0.9642	0.7147	3.5714
D2S1338	0.8500	0.8809	0.7905	0.8641	0.9672	0.6949	3.3333
Penta D	0.7700	0.8267	0.2808	0.8010	0.9420	0.5446	2.1739

Supplementary Table S6. Forensic parameters for 23 STRs in Tibetan population (N=100).

Locus	H_o	H_e	p-value	PIC	PD	PE	TPI
D3S1358	0.7200	0.7504	0.2115	0.7028	0.8872	0.4599	1.7857
vWA	0.8000	0.7875	0.8087	0.7505	0.9142	0.5990	2.5000
D16S539	0.7400	0.78820	0.0264	0.7539	0.9136	0.4928	1.9231
CSF1PO	0.7100	0.7147	0.9431	0.6653	0.8708	0.4439	1.7241
TPOX	0.6600	0.6450	0.4798	0.5732	0.7950	0.3691	1.4706
D8S1179	0.8200	0.8327	0.1298	0.8067	0.9350	0.6367	2.7778
D21S11	0.8000	0.8466	0.2002	0.8260	0.9512	0.5990	2.5000
D18S51	0.8700	0.8600	0.4048	0.8413	0.9564	0.7346	3.8462
Penta E	0.9100	0.9262	0.4601	0.9161	0.9798	0.8159	5.5556
D2S441	0.7400	0.7623	0.1567	0.7184	0.8934	0.4928	1.9231
D19S433	0.8500	0.8441	0.7552	0.8206	0.9484	0.6949	3.3333
TH01	0.5900	0.6463	0.2652	0.5857	0.8262	0.2791	1.2195
FGA	0.8900	0.8750	0.1832	0.8573	0.9596	0.7750	4.5455
D22S1045	0.7500	0.7635	0.0904	0.7180	0.8928	0.5098	2.0000
D5S818	0.6900	0.7353	0.4341	0.6898	0.8892	0.4130	1.6129
D13S317	0.8900	0.8206	0.8917	0.7913	0.9334	0.7750	4.5455
D7S820	0.7700	0.7854	0.7776	0.7513	0.9154	0.5625	2.2727
D6S1043	0.8900	0.8789	0.9288	0.8617	0.9654	0.7750	4.5455
D10S1248	0.7500	0.7502	0.6822	0.7060	0.8900	0.5098	2.0000
D1S1656	0.8300	0.8143	0.1687	0.7873	0.9238	0.6559	2.9412
D12S391	0.8800	0.8439	0.6707	0.8251	0.9472	0.7548	4.1667
D2S1338	0.8700	0.8526	0.2697	0.8309	0.9510	0.7346	3.8462
Penta D	0.7500	0.8242	0.6287	0.7963	0.9424	0.5098	2.0000

Supplementary Table S7. p-values of LD test between 23 STR loci from HAN.

Loci	L1	L2	L3	L4	L5	L6	L7	L8	L9	L10	L11	L12	L13	L14	L15	L16	L17	L18	L19	L20	L21	L22
L1																						
L2	0.0723																					
L3	0.1046	0.7781																				
L4	0.2463	0.7340	0.9951																			
L5	0.9990	0.7742	0.1408	0.2092																		
L6	0.7058	0.6070	0.6139	0.3490	0.6706																	
L7	0.3578	0.6823	0.0792	0.0645	0.8113	0.8074																
L8	0.7967	0.4067	0.9785	0.7331	0.0723	0.0176	0.9961															
L9	0.0127	0.5044	0.3881	0.7155	0.2581	0.5034	0.8514	0.3656														
L10	0.8475	0.0978	0.4223	0.5936	0.9032	0.3206	0.6725	0.2493	0.9228													
L11	0.5894	0.0831	0.8974	0.8153	0.7771	0.9844	0.0264	0.1320	0.7312	0.6569												
L12	0.4145	0.5005	0.2063	0.2063	0.1203	0.4846	0.8397	0.2874	0.3206	0.7380	0.3763											
L13	0.0098	0.9785	0.5415	0.4213	0.0440	0.9971	0.5523	0.9863	0.1095	0.0929	0.3881	0.5532										
L14	0.0489	0.6383	0.4487	0.0518	0.6637	0.9169	0.2170	0.1926	0.4213	0.9462	0.3822	0.0880	0.1369									
L15	0.3910	0.8358	0.8954	0.1848	0.4076	0.5200	0.8338	0.8592	0.5142	0.6862	0.1261	0.9873	0.7449	0.3831								
L16	0.9531	0.9286	0.3529	0.0225	0.5601	0.5679	0.0127	0.9844	0.6725	0.1994	0.0899	0.6540	0.9785	0.7713	0.0489							
L17	0.4487	0.5103	0.1867	0.2806	0.9502	0.8104	0.3363	0.8573	0.3822	0.8123	0.7957	0.2454	0.3304	0.8211	0.9159	0.7341						
L18	0.3167	0.4800	0.6373	0.1007	0.9042	0.0753	0.2913	0.8534	0.9198	0.1290	0.4311	0.8504	0.8397	0.9717	0.5415	0.3666	0.8025					
L19	0.4291	0.6980	0.7791	0.4897	0.1818	0.2669	0.9941	0.6373	0.8935	0.9150	0.2942	0.4027	0.3754	0.5151	0.1691	0.9130	0.8182	0.4037				
L20	0.2004	0.2092	0.4174	0.1857	0.1075	0.7781	0.9893	0.1486	0.8915	0.3773	0.8006	0.2287	0.0323	0.0450	0.6637	0.7302	0.1300	0.6921	0.3900			
L21	0.6755	0.7937	0.6628	0.0929	0.3588	0.6637	0.6774	0.3744	0.7937	0.4311	0.6481	0.2454	0.4223	0.2258	0.6080	0.0508	0.1760	0.8113	0.8407	0.5152		
L22	0.1036	0.2317	0.0284	0.8006	0.8886	0.7341	0.4242	0.4927	0.1339	0.8651	0.2444	0.0792	0.7234	0.8358	0.6755	0.4839	0.6061	0.4233	0.6413	0.0587	0.3793	
L23	0.5112	0.8123	0.1085	0.5572	0.1476	0.3979	0.2004	0.5161	0.4467	0.8983	0.9726	0.1681	0.9218	0.6950	0.2317	0.7136	0.6540	0.2072	0.3069	0.5210	0.1818	0.3910

Supplementary Table S8. p-values of LD test between 23 STR loci from Uygur.

Loci	L1	L2	L3	L4	L5	L6	L7	L8	L9	L10	L11	L12	L13	L14	L15	L16	L17	L18	L19	L20	L21	L22
L1																						
L2	0.4868																					
L3	0.3216	0.9795																				
L4	0.4995	0.0753	0.5865																			
L5	0.3392	0.0401	0.8289	0.4008																		
L6	0.7439	0.6139	0.1906	0.5914	0.6755																	
L7	0.4027	0.4360	0.7253	0.9834	0.5523	0.3333																
L8	0.1281	0.8299	0.3959	0.4878	0.5582	0.8661	0.3803															
L9	0.9169	0.9443	0.6452	0.4242	0.2395	0.6334	0.3646	0.3128														
L10	0.4184	0.0694	0.3744	0.4135	0.3842	0.1193	0.7204	0.4927	0.4311													
L11	0.2327	0.1740	0.0166	0.2854	0.1007	0.8123	0.6305	0.1486	0.5005	0.5543												
L12	0.2121	0.2434	0.3372	0.9140	0.1928	0.9443	0.9267	0.8710	0.3402	0.3959	0.5386											
L13	0.9746	0.8544	0.6031	0.7625	0.6970	0.1046	0.2473	0.8309	0.5767	0.7009	0.8720	0.7146										
L14	0.3245	0.7732	0.0978	0.6637	0.3724	0.1760	0.4682	0.4878	0.9580	0.3030	0.9189	0.4057	0.7899									
L15	0.4125	0.1388	0.0792	0.6598	0.9912	0.6481	0.1984	0.5865	0.7732	0.8094	0.2033	0.5807	0.3500	0.5240								
L16	0.9726	0.4409	0.3216	0.1887	0.4633	0.7361	0.7204	0.3480	0.3861	0.4301	0.3636	0.9169	0.2727	0.6725	0.6618							
L17	0.3157	0.4477	0.1359	0.2033	0.7341	0.1212	0.4731	0.1662	0.9482	0.1212	0.6882	0.9990	0.8710	0.0675	0.2053	0.5582						
L18	0.1026	0.7674	0.2845	0.8592	0.2199	0.3304	0.5946	0.3861	0.2581	0.3988	0.5610	0.9648	0.9453	0.2277	0.6696	0.5200	0.7077					
L19	0.5982	0.8739	0.9873	0.1300	0.2375	0.7742	0.1134	0.4780	0.3509	0.7116	0.2121	0.1486	0.4536	0.5904	0.3285	0.4379	0.4409	0.3666				
L20	0.5376	0.1466	0.1691	0.4555	0.2727	0.6657	0.5748	0.1926	0.5894	0.2806	0.9189	0.5826	0.8495	0.5767	0.9277	0.9384	0.4379	0.5963	0.9824			
L21	0.0831	0.4418	0.7175	0.6266	0.7155	0.8886	0.9589	0.4565	0.8602	0.9756	0.1672	0.6794	0.8172	0.0196	0.5777	0.4839	0.9267	0.2297	0.7243	0.1066		
L22	0.7273	0.1984	0.8837	0.0743	0.7947	0.0684	0.7273	0.6999	0.5142	0.9218	0.7459	0.8768	0.5689	0.3382	0.1290	0.7595	0.5445	0.8084	0.5396	0.0538	0.1300	
L23	0.5572	0.8983	0.4976	0.9609	0.7507	0.3480	0.7380	0.4370	0.1095	0.8680	0.7762	0.9580	0.1144	0.4418	0.1525	0.4692	0.8319	0.4027	0.4194	0.1320	0.6980	0.0821

Supplementary Table S9. p-values of LD test between 23 STR loci from Tibetan.

Loci	L1	L2	L3	L4	L5	L6	L7	L8	L9	L10	L11	L12	L13	L14	L15	L16	L17	L18	L19	L20	L21	L22
L1																						
L2	0.4712																					
L3	0.2004	0.5210																				
L4	0.5034	0.0352	0.0117																			
L5	0.9863	0.7527	0.4233	0.5963																		
L6	0.2102	0.3324	0.6041	0.8534	0.4516																	
L7	0.0049	0.6266	0.5513	0.6198	0.6560	0.2952																
L8	0.9658	0.5865	0.2444	0.1232	0.7488	0.3793	0.1359															
L9	0.1554	0.4428	0.2718	0.2669	0.2121	0.4184	0.5513	0.5826														
L10	0.0587	0.2786	0.3118	0.0547	0.9707	0.1652	0.3089	0.8749	0.3949													
L11	0.2933	0.0714	0.7674	0.4330	0.6569	0.8915	0.0723	0.9941	0.7488	0.6559												
L12	0.1789	0.7996	0.1486	0.8817	0.1965	0.7683	0.7214	0.1095	0.0567	0.6237	0.0205											
L13	0.3607	0.1877	0.9589	0.7419	0.8895	0.6393	0.8485	0.3500	0.6764	0.3372	0.5210	0.7429										
L14	0.1095	0.6589	0.9198	0.0606	0.9472	0.5855	0.7028	0.2160	0.3089	0.0117	0.1212	0.7898	0.3500									
L15	0.7331	0.2962	0.9032	0.1300	0.3470	0.8768	0.7664	0.8915	0.1545	0.4203	0.4506	0.3285	0.8182	0.4829								
L16	0.8485	0.0880	0.6070	0.5337	0.0763	0.2806	0.2991	0.9013	0.7605	0.4546	0.5787	0.3490	0.1623	0.3060	0.5191							
L17	0.5240	0.8847	0.3021	0.0850	0.5494	0.6882	0.4282	0.6237	0.7576	0.5543	0.4702	0.2121	0.4213	0.0567	0.3480	0.6931						
L18	0.1085	0.5963	0.1720	0.8113	0.8025	0.3773	0.4252	0.2845	0.7028	0.6061	0.2258	0.2297	0.0362	0.7996	0.9775	0.6989	0.2121					
L19	0.2913	0.8661	0.5591	0.2972	0.6168	0.8632	0.4428	0.4106	0.0880	0.1183	0.6569	0.1975	0.6256	0.7038	0.2102	0.8201	0.4721	0.6334				
L20	0.2297	0.6129	0.6207	0.3187	0.7859	0.1691	0.5044	0.3353	0.8348	0.2004	0.8084	0.2063	0.4027	0.4458	0.7116	0.7654	0.2082	0.2385	0.0029			
L21	0.9013	0.4878	0.6735	0.7028	0.0645	0.3285	0.9990	0.3236	0.0841	0.6452	0.1984	0.4125	0.9570	0.2014	0.1056	0.6012	0.3030	0.7341	0.7937	0.6696		
L22	0.9042	0.8602	0.6246	0.9541	0.9032	0.2532	0.2297	0.1652	0.4330	0.3138	0.4839	0.3441	0.5904	0.3539	0.1437	0.2894	0.3627	0.0108	0.9932	0.0626	0.4360	
L23	0.3636	0.6315	0.1574	0.8964	0.6315	0.8680	0.7165	0.8690	0.7517	0.6735	0.7517	0.8983	0.3480	0.5396	0.1046	0.9013	0.7957	0.1369	0.3676	0.3910	0.9619	0.8661

Supplementary Table S10. Allelic frequencies of 23 STRs in 3 main ethnic groups of China.

Locus	Allele	HAN (n=202)	Uygur (n=100)	Tibetan (n=100)	Locus	Allele	HAN (n=202)	Uygur (n=100)	Tibetan (n=100)
**D3S1358**	11			0.0050	**vWA**	13	0.0025	0.0100	
	13		0.0050	0.0150		14	0.2302	0.1700	0.2150
	14	0.0446	0.0900	0.0350		15	0.0198	0.0650	0.0350
	15	0.3144	0.2800	0.3200		16	0.1955	0.1850	0.2000
	16	0.3589	0.2900	0.2700		17	0.2574	0.2300	0.3100
	17	0.2079	0.2000	0.2600		18	0.2030	0.2350	0.1650
	18	0.0619	0.1200	0.0950		19	0.0792	0.0850	0.0750
	19	0.0099	0.0150			20	0.0124	0.0200	
	20	0.0025			**CSF1PO**	7	0.0050		
**D16S539**	8	0.0099	0.0500	0.0300		8	0.0025		
	9	0.3267	0.1800	0.2150		9	0.0569	0.0350	0.0300
	10	0.0941	0.1500	0.1250		10	0.2376	0.3100	0.2300
	11	0.2030	0.2750	0.3350		11	0.2450	0.2150	0.2150
	12	0.2129	0.2250	0.1850		12	0.3787	0.3650	0.4250
	13	0.1386	0.1050	0.0750		13	0.0594	0.0550	0.0900
	14	0.0149	0.0150	0.0300		14	0.0149	0.0200	0.0100
	15			0.0050	**D8S1179**	8		0.0050	
**TPOX**	8	0.5297	0.4850	0.4600		10	0.1188	0.1100	0.1050
	9	0.1337	0.1100	0.1650		11	0.0891	0.0650	0.0350
	10	0.0248	0.0750	0.0100		12	0.1262	0.1150	0.1000
	11	0.2722	0.2850	0.3450		13	0.2178	0.2400	0.2600
	12	0.0347	0.0450	0.0150		14	0.1980	0.2300	0.1800
	13			0.0050		15	0.1609	0.1600	0.2050
**D21S11**	26	0.0025				16	0.0743	0.0600	0.0750
	28	0.0495	0.0950	0.0350		17	0.0124	0.0150	0.0400
	28.2	0.0099	0.0100	0.0400		18	0.0025		
	29	0.2995	0.2350	0.2000	**D18S51**	10		0.0150	
	29.2	0.0025		0.0050		10.2		0.0050	
	30	0.2748	0.2450	0.2850		12	0.0222	0.0700	0.0200
	30.2	0.0074	0.0300	0.0500		13	0.1807	0.1600	0.2700
	30.3	0.0149	0.0050			14	0.2574	0.1850	0.1650
	31	0.0792	0.0800	0.0750		15	0.1807	0.1500	0.1100
	31.2	0.0668	0.1050	0.0800		16	0.1188	0.1200	0.1050
	32	0.0396	0.0250	0.0200		17	0.0866	0.1550	0.0850
	32.2	0.1089	0.1350	0.1050		18	0.0396	0.0650	0.0650
	33	0.0025				19	0.0222	0.0150	0.0800
	33.2	0.0347	0.0300	0.0850		20	0.0371	0.0200	0.0300
	34.1	0.0025				21	0.0099	0.0200	0.0400
	34.2			0.0200		22	0.0356		0.0150
	35.2	0.0050	0.0050			23	0.0099	0.0200	0.0050

**Penta E**	5	0.0371	0.0650	0.0550
	7	0.0025	0.0550	0.0100
	8	0.0050	0.0150	0.0100
	9	0.0074	0.0100	0.0100
	10	0.0470	0.0750	0.0250
	11	0.1361	0.0700	0.1000
	12	0.1188	0.1050	0.1200
	13	0.0420	0.0550	0.0250
	14	0.0693	0.0350	0.0700
	15	0.1139	0.0900	0.1100
	16	0.0990	0.1150	0.1100
	16.4		0.0100	
	17	0.1015	0.0700	0.0750
	18	0.0594	0.0800	0.0750
	18.4	0.0025		
	19	0.0545	0.0800	0.0550
	19.4	0.0025		
	20	0.0421	0.0450	0.0550
	21	0.0222	0.0050	0.0350
	22	0.0173	0.0150	0.0400
	23	0.0099	0.0050	0.0150
	24	0.0074		0.0050
	25	0.0025		
**D19S433**	10		0.0050	
	11		0.0050	
	12	0.0371	0.0600	0.0350
	12.2	0.0050		
	13	0.2624	0.2300	0.2450
	13.2	0.0495	0.0550	0.0550
	14	0.2500	0.2900	0.2050
	14.2	0.1163	0.0900	0.1400
	15	0.0644	0.1050	0.1050
	15.2	0.1633	0.0950	0.1450
	16	0.0124	0.0250	0.0350
	16.2	0.0396	0.0350	0.0300
	17		0.0050	0.0050
**D22S1045**	11	0.2599	0.2450	0.3050
	12	0.0050		
	13	0.0050		
	14	0.0124	0.0450	0.0250
	15	0.3069	0.3200	0.2000
	16	0.2500	0.2750	0.2500
	17	0.1361	0.1050	0.2100
	18	0.0222	0.0100	0.0100
	24			0.0050
	25	0.0025		
	26	0.0025		0.0050
**D2S441**	8.1	0.0025		
	9.1	0.0297	0.0200	0.0050
	10	0.2376	0.1950	0.2900
	11	0.3020	0.3650	0.3050
	11.3	0.0347	0.0300	
	12	0.1757	0.1300	0.1150
	12.3	0.0050		
	13	0.0248	0.0400	0.0500
	14	0.1733	0.1850	0.2200
	15	0.0124	0.0250	0.0150
	16	0.0020	0.0100	
**TH01**	6	0.1188	0.1600	0.0300
	7	0.2525	0.2700	0.3150
	8	0.0545	0.12500	0.0600
	9	0.5099	0.3000	0.4950
	9.3	0.0471	0.1400	0.0900
	10	0.0149	0.0050	0.0050
	11	0.0025		0.0050
**FGA**	14	0.0025		
	16	0.0025		
	18	0.0347	0.0050	0.0850
	19	0.0297	0.0300	0.0450
	20	0.0272	0.0850	0.0250
	21	0.1188	0.1200	0.0550
	21.2	0.0050		
	22	0.1436	0.2050	0.1450
	22.1			0.0050
	22.2	0.0198	0.0050	0.0100
	23	0.2450	0.1950	0.1850
	23.2			0.0050
	24	0.1906	0.2150	0.1950
	24.2	0.0050	0.0050	0.0350
	25	0.0866	0.1050	0.1300
	25.2	0.0099		
	26	0.0644	0.0150	0.0700
	26.2	0.0025		0.0050
	27	0.0124	0.0150	0.0050
**D5S818**	7	0.0272	0.0200	0.0200
	8	0.0074		
	9	0.0767	0.0700	0.0350
	10	0.1485	0.1050	0.1650

	19	0.0050		
**D13S317**	5			0.0100
	7		0.0050	
	8	0.2723	0.2050	0.2600
	9	0.1361	0.1000	0.0850
	10	0.1312	0.1200	0.1550
	11	0.2500	0.3100	0.1850
	12	0.1436	0.2000	0.2150
	13	0.0470	0.0400	0.0500
	14	0.0198	0.0200	0.040
**D6S1043**	7		0.0100	
	8		0.0100	0.0150
	10	0.0248	0.0150	0.0350
	11	0.1089	0.2250	0.1600
	12	0.1361	0.1300	0.1650
	13	0.1139	0.1250	0.1300
	14	0.1460	0.0800	0.0900
	15	0.0149	0.0150	0.0100
	17	0.0495	0.0300	0.0550
	18	0.2153	0.1950	0.1800
	19	0.1213	0.0900	0.0950
	20	0.0470	0.0550	0.0300
	20.3	0.0025		0.010
	21	0.0149	0.0100	
	21.3	0.0025	0.0050	0.0250
	22		0.0050	
	22.3	0.0025		
**D12S391**	15	0.0149	0.0200	0.0300
	16	0.0050	0.0100	0.0150
	17	0.1015	0.1250	0.1350
	17.3		0.0050	0.0050
	18	0.2525	0.1750	0.2450
	18.3		0.0150	
	19	0.2129	0.1800	0.2050
	19.3		0.0100	
	20	0.1584	0.1750	0.1650
	21	0.1015	0.1050	0.0700
	22	0.0891	0.0550	0.0600
	23	0.0446	0.0850	0.0550
	24	0.0149	0.0250	0.0100
	25	0.0050	0.0100	
	26		0.0050	0.0050
**Penta D**	6		0.0150	0.0100
	7		0.0050	0.0050
	11	0.3069	0.3050	0.3950
	12	0.2822	0.3200	0.2650
	13	0.1411	0.1700	0.1150
	14	0.0099	0.0100	0.0050
**D7S820**	7	0.0025	0.0200	
	8	0.1436	0.2300	0.1950
	9	0.0619	0.0800	0.0750
	9.1	0.0050		
	10	0.1683	0.1900	0.1300
	10.1	0.0025	0.2600	0.0050
	11	0.3540	0.1750	0.2700
	12	0.2178	0.0400	0.2900
	13	0.0446	0.0050	0.0300
	14			0.0050
**D10S1248**	8	0.0025	0.0050	
	11	0.0025	0.0050	0.0050
	12	0.0817	0.0250	0.0600
	13	0.4109	0.2850	0.3650
	14	0.2426	0.2900	0.2400
	15	0.1832	0.2250	0.2300
	16	0.0619	0.1200	0.0750
	17	0.0149	0.0400	0.0250
	18		0.0050	
**D1S1656**	8		0.0050	
	9		0.0050	
	11	0.0396	0.0550	0.0200
	12	0.0421	0.0650	0.0750
	13	0.0693	0.0600	0.1200
	14	0.0718	0.0950	0.0650
	14.3		0.0050	
	15	0.3540	0.2750	0.3050
	15.3	0.0074		0.0150
	16	0.2450	0.2000	0.2550
	16.3	0.0025	0.0200	0.0150
	17	0.0719	0.1050	0.0750
	17.3	0.0569	0.0600	0.0200
	18	0.0074	0.0100	0.0200
	18.3	0.0272	0.0300	0.0150
	19	0.0025		
	19.3	0.0025	0.0100	
**D2S1338**	16	0.0124	0.0050	0.0100
	17	0.0569	0.1150	0.0300
	18	0.1064	0.1150	0.0700
	19	0.1535	0.1750	0.2350

8	0.0594	0.0300	0.0500	20	0.1213	0.0950	0.1800
9	0.3243	0.3000	0.2550	21	0.0347	0.0200	0.0700
10	0.1114	0.1300	0.1250	22	0.0767	0.0450	0.0400
11	0.1535	0.1450	0.2400	23	0.2054	0.1900	0.1850
12	0.1535	0.1550	0.1600	24	0.1361	0.0950	0.1250
13	0.1535	0.1450	0.1100	25	0.0792	0.0850	0.0400
14	0.0371	0.0550	0.0350	26	0.0099	0.0500	0.0150
15	0.0074	0.0150	0.0100	27	0.0050		
16		0.0050		28	0.0025	0.0100	

Supplementary Table S11. p-values of population differentiation exact test between HAN and the other 2 ethnic groups

STR loci	Uygur	Tibetan
D3S1358	0.7470 ± 0.0035	0.3643 ±0 .0148
vWA	0.6741 ± 0.0033	0.8303 ± 0.0075
D16S539	0.4038 ± 0.0030	0.0152 ± 0.0017
CSF1PO	0.9436 ± 0.0027	0.9004 ± 0.0050
TPOX	0.5588 ± 0.0064	0.2665 ± 0.0116
D8S1179	0.8540 ± 0.0101	0.6478 ± 0.0108
D21S11	0.8662 ± 0.0083	0.0719 ± 0.0086
D18S51	0.6846 ± 0.0130	0.0466 ± 0.005
Penta E	0.7326 ± 0.0106	0.9884 ± 0.0018
D2S441	0.9655 ± 0.0028	0.3541 ± 0.0095
TH01	0.4718 ± 0.0111	0.0924 ± 0.0053
D19S433	0.7963 ± 0.0056	0.7185 ± 0.0128
FGA	0.9384 ± 0.0064	0.2234 ± 0.0120
D22S1045	0.7217 ± 0.0178	0.38190 ± 0.0140
D5S818	0.9904 ± 0.0009	0.8242 ± 0.0107
D13S317	0.9819 ± 0.0009	0.3451 ± 0.0108
D7S820	0.0022 ± 0.0007	0.4805 ± 0.0197
D6S1043	0.9750 ± 0.0027	0.5781 ± 0.0107
D10S1248	0.8058 ± 0.0074	0.8412 ± 0.0093
D12S391	0.9689 ± 0.0020	0.7654 ± 0.0129
D1S1656	0.7952 ± 0.0079	0.6904 ± 0.0168
D2S1338	0.7599 ± 0.0076	0.5188 ± 0.0140
Penta D	0.1808 ± 0.0085	0.3806 ± 0.0121
